# Exploratory Multi-Compartment Profiling of Steroid and Bile Acid Metabolites in Relation to Maternal Perceived Stress at Term

**DOI:** 10.3390/cells15211982

**Published:** 2026-10-30

**Authors:** Raquel Gomez Junco Lobo, Akshay Uttarkar, Jeannette Lager, Aditi Bhargava

**Affiliations:** 1 Aseesa Inc., South San Francisco, CA 94080, USA; raquelgomezjunco@gmail.com (R.G.J.L.); akshay@aseesa.com (A.U.); 2 Department of ObGyn, University of California San Francisco, San Francisco, CA 94143, USA; jeannette.lager@ucsf.edu

**Keywords:** bile acids, cord plasma, centering pregnancy, estrone, maternal hair, mass spectrometry

## Abstract

**Highlights:**

**What are the main findings?**

- Report of bile acid and steroid metabolites across four maternal–fetal compartments. Hair metabolite levels did not track plasma or placental directionality well.
- Most steroid and bile acid metabolites remained relatively stable across compartments and displayed no significant association with PSS, sociodemographic scores, or gestational diabetes, in the primary or secondary analyses.

**What are the implications of the main findings?**

- Findings are consistent with placental buffering of maternal perceived and sociodemographic stressors.
- E1, 20-OH-PROG, DHP, PELN, GCDCA, and UDCA are exploratory, hypothesis-generating signals, and warrant testing in larger, adequately powered cohorts.

**Abstract:**

Maternal stressors during pregnancy are linked to adverse birth outcomes, but the biochemical pathways involved remain poorly defined. We previously reported that the human placenta buffers the fetus from several effects of maternal perceived stress in a cohort of term pregnancies. Here, using the same cohort and no additional participants, we analyze a broader steroid panel and, for the first time, a targeted bile acid metabolomic panel across four maternal–fetal compartments: maternal plasma, placental tissue, cord plasma, and maternal hair. Nulliparous women were recruited during the third trimester at UCSF Mission Bay Hospital; perceived stress was measured using the 10-item Perceived Stress Scale (PSS) and retained as a continuous variable for the primary analysis from 17 women. Of 212 analyte compartment pairs evaluated, 38 metabolites met a predefined detectability criterion and were tested for association with PSS and sociodemographic scores using Kendall’s tau b, with permutation *p*-values, bootstrap confidence intervals, and Benjamini–Hochberg correction for multiple comparisons. No association survived multiple testing corrections. In exploratory, uncorrected comparisons (*p* < 0.05), three metabolites were nominally lower in a cohort-derived, higher-PSS group: placental estrone (E1), cord plasma glycochenodeoxycholic acid (GCDCA), and maternal plasma ursodeoxycholic acid (UDCA). Nearly 47.1% of women had gestational diabetes; 20-hydroxyprogesterone (20-OH-PROG) in maternal plasma, dihydroprogesterone (DHP), and pregnenolone (PELN) in placental tissues were nominally decreased in women who developed gestational diabetes compared to women who did not develop gestational diabetes. Maternal hair was not a good compartment for detection of bile acid metabolites. Despite a wide range of perceived stress/sociodemographic scores and a substantial burden of maternal clinical comorbidities in this cohort, all pregnancies resulted in favorable neonatal outcomes at delivery. These findings support the hypothesis that the placenta functions as a buffering organ that protects the developing fetus against broad insults while remaining susceptible to more selective disruptions.

## 1. Introduction

### 1.1. Maternal Stress and Pregnancy Outcomes

The placenta is a temporary organ that develops in the uterus during pregnancy. It connects the developing fetus via the umbilical cord and attaches to the uterine wall, facilitating nutrient exchange, oxygen transport, waste removal, and endocrine signaling between pregnant women and the fetus. Beyond its vascular functions, the placenta also serves as a complex endocrine and immune organ that supports fetal growth, regulates pregnancy-related physiology in females, and protects the fetus from harmful environmental exposures and infections. The human placenta takes over the primary production of progesterone and estrogen from the ovaries around the first 9 weeks of gestation. These include hormones such as estrone (E1), estradiol (E2), and estriol (E3), which play essential roles in maintaining pregnancy, regulating placental function, and supporting fetal development [1,2,3]. Physiological concentrations of estradiol in human pregnancy range from 0.15 to 3.34 ng/mL in the 1st trimester, from 1.56 to 21.28 ng/mL in the 2nd trimester, and from 8.85 to ≥30.0 ng/mL in the 3rd trimester. Whereas estradiol levels fall below ≤0.02 ng/mL in the weeks after birth [4].

Although estrogens are frequently grouped in clinical and research settings, E1, E2, and E3 possess distinct biological roles and metabolic pathways. However, little is known about whether maternal stress selectively alters specific estrogen metabolites across maternal and fetal compartments. Progesterone and estrogen signaling are critical for maintaining a healthy pregnancy and supporting fetal development. Progesterone concentrations rise steadily throughout gestation and are essential for maintaining uterine quiescence, modulating maternal immune tolerance, and supporting placental function.

Longitudinal hormonal profiling has demonstrated that both progesterone and E2 increase substantially across pregnancy before rapidly declining postpartum, reflecting major endocrine adaptations required to sustain gestation [4]. Estrogen metabolites also increase throughout pregnancy and contribute to placental growth, fetal organ maturation, and preparation for labor. Dysregulation of these hormonal pathways has been associated with adverse pregnancy outcomes, including preterm birth (before 37 weeks of gestation age) and fetal growth restriction. A full-term human pregnancy encompasses gestational age ranging from 39 weeks, 0 days (39 + 0) to 40 weeks, 6 days (Figure S1). Studies have identified distinct plasma metabolomic signatures in women with preeclampsia and preterm delivery, including disruptions in lipid metabolism and inflammatory pathways associated with long-term maternal cardiovascular risk [5]. Despite their biological importance, the relationship between maternal perceived stress and compartment-specific steroid metabolite profiles remains poorly understood.

Maternal psychological stress during pregnancy, driven by various factors like socioeconomic disparities, trauma, or physiological and psychosocial stressors, has been associated with adverse maternal and neonatal outcomes, including preterm birth, low birth weight, and altered neurodevelopmental trajectories in offspring. Psychological stress during pregnancy is common, with a substantial proportion of pregnant individuals reporting significant life stressors during gestation. Nearly three-quarters of pregnant individuals report experiencing at least one significant stressor in the year before delivery, highlighting the widespread prevalence of stress during pregnancy and the importance of understanding its biological consequences [6]. In metabolomic studies of pregnant individuals with depression, significant alterations in amino acid, lipid, and energy metabolism pathways have been observed during pregnancy and postpartum, suggesting that psychological stress may produce measurable biochemical changes across multiple metabolic systems [7]. To measure this stress, the Perceived Stress Scale (PSS) is used because it is the most widely used psychological instrument for measuring the perception of stress. Created by Sheldon Cohen and colleagues in 1983, it evaluates how unpredictable, uncontrollable, and overloaded individuals find their lives [8,9].

Although cortisol has frequently been used as a biomarker of stress during pregnancy, studies examining the relationship between cortisol, perceived stress, and adverse birth outcomes have produced inconsistent findings, suggesting that additional biomarkers may be needed to characterize stress-related physiological changes during pregnancy. The placenta plays a critical role in protecting the fetus from excessive maternal stress signaling by regulating the transfer and metabolism of glucocorticoids and steroid hormones. As part of the maternal–fetal stress response system, the placenta responds dynamically to maternal cortisol levels and contributes to the development of the fetal hypothalamic–pituitary–adrenal (HPA) axis. Excessive or chronic maternal stress may disrupt placental endocrine and metabolic pathways, potentially altering steroid hormone synthesis and fetal exposure to bioactive metabolites. However, the biochemical mechanisms linking maternal perceived stress to altered pregnancy outcomes remain incompletely understood. One of the primary biological pathways linking maternal stress to fetal outcomes involves activation of the HPA axis, which alters circulating endocrine and metabolic signaling during pregnancy. Activation of the maternal HPA axis increases glucocorticoid signaling and may alter downstream steroidogenesis and metabolic pathways within the placenta [10]. Because the placenta functions as a major endocrine organ throughout gestation, stress-associated hormonal changes may influence placental metabolism and fetal exposure to bioactive metabolites. The placenta naturally acts as a protective barrier that shields the fetus from excess maternal hormones, functioning not just as a physical interface but as an active biochemical regulator of fetal exposure to stress-associated metabolites. Chronic or excessive maternal stress can compromise this buffering capacity, potentially altering fetal brain development, endocrine regulation, and stress reactivity later in life.

Yet despite how common both psychological stress and clinical comorbidity are during pregnancy, most pregnancies still culminate in a relatively healthy birth. This gap between exposure and outcome is itself informative: it suggests that maternal-fetal physiology includes mechanisms (placenta) that absorb a substantial degree of maternal adversity before it manifests as measurable harm to the fetus, rather than adversity translating directly into poor outcomes in a linear, dose-dependent way. Characterizing the biochemical signature of that buffering, and identifying which insults perturb this buffering capacity, is the motivation for the present study. Here, we tested if insults such as perceived stress and/or socioeconomic status, experienced by women worldwide, influence specific or global bile acid and steroid metabolite signatures.

### 1.2. Centering Pregnancy vs. Individual Prenatal Care

Centering Pregnancy is a group-based prenatal care model that replaces traditional 15 min doctor visits with 90 min to 2 h group sessions. Groups of 8–12 expectant women with similar gestational ages meet with healthcare providers throughout their pregnancy to check vitals, receive prenatal education, and receive peer support. This is different from the traditional one-on-one appointments. Centering Pregnancy has been associated in some studies with improved prenatal education, increased patient engagement, and reduced risk of adverse birth outcomes, potentially through enhanced social support and stress reduction [11]. UCSF Mission Bay Hospital offered both Centering and individual prenatal care; participants came from both settings.

### 1.3. Bile Acids in Pregnancy and Association with Gestational Diabetes Mellitus

Bile acids are synthesized in the liver from cholesterol. Primary bile acids, including cholic acid (CA) and chenodeoxycholic acid (CDCA), act as signaling molecules through receptors such as FXR and TGR5, regulating glucose homeostasis, inflammation, lipid metabolism, and endocrine signaling [12], and may be converted into secondary bile acids through intestinal microbial metabolism. Conjugated bile acids, including glycochenodeoxycholic acid (GCDCA) and unconjugated ursodeoxycholic acid (UDCA), participate in lipid digestion, metabolic and inflammatory regulation.

Altered bile acid homeostasis during pregnancy has been associated with adverse maternal and fetal outcomes, yet its relationship with maternal psychological stress remains poorly understood. In pregnancy, bile acid metabolism shifts significantly; elevated bile acids are the hallmark of intrahepatic cholestasis of pregnancy (ICP), associated with preterm birth and stillbirth. Certain bile acids, including ursodeoxycholic acid (UDCA), have also been investigated therapeutically in ICP because of their anti-inflammatory and hepatoprotective effects. Studies have reported that UDCA therapy reduced serum bile acid concentrations and improved liver function markers in patients with intrahepatic cholestasis of pregnancy [13]. Systematic metabolomic analyses of ICP pregnancies have demonstrated widespread alterations in both bile acid and steroid metabolic pathways, suggesting close biochemical interactions between hepatic function and endocrine regulation during pregnancy [14]. Despite growing recognition of the importance of bile acid signaling during pregnancy, the extent to which maternal stress influences bile acid metabolism across maternal and fetal compartments remains largely unexplored.

Gestational diabetes mellitus (GDM) is associated with increased risk of preterm birth and macrosomia in women aged ≤ 35 years [15,16] and other adverse outcomes such as preeclampsia and neonatal ICU (NICU) admissions [16]. In a case–control study, higher serum levels of corticosteroids, progestins, and estrogen metabolites were reported in women with GDM compared with healthy pregnant women who did not develop GDM [17]. In other observational studies, elevated levels of bile acid metabolites were reported before women developed GDM [18], whereas others have suggested a link between DM and lithocholic acid (LCA) and β-muricholic acid (β-MCA) [19]. Bile acids and steroid metabolites can both regulate glucose and lipid metabolism via activating the Farnesoid X receptor (FXR) and Takeda G protein-coupled receptor 5 (TGR5). Nearly 47.1% of the women in our cohort developed GDM, prompting us to evaluate the association of steroid and bile acid metabolites with GDM.

Although maternal stress has been associated with adverse pregnancy outcomes, little is known about how perceived stress alters compartment-specific steroid and bile acid metabolism across the maternal–fetal interface. Most prior studies have focused on isolated hormones or single biospecimens, leaving the broader metabolomic response to maternal stress poorly characterized. Previous metabolomic investigations of pregnancy complications have primarily relied on maternal plasma samples, with few studies simultaneously evaluating maternal, placental, fetal, and noninvasive biospecimens across the maternal–fetal interface [5,7]. This paper provides the first systematic description of bile acid metabolites across all four compartments: maternal plasma, cord plasma, placental tissue, and maternal hair in a cohort of term pregnancies. We further evaluated whether maternal perceived stress, measured using the PSS-10 questionnaire, was associated with alterations in specific metabolites across maternal and fetal compartments. Finally, we explored whether maternal hair metabolites reliably reflected plasma metabolite levels as a potential noninvasive biomarker approach. By integrating steroid and bile acid metabolomics across multiple maternal–fetal compartments, this study seeks to provide new insight into the biochemical pathways associated with maternal perceived stress during pregnancy. Simultaneous analysis of maternal plasma, cord plasma, placental tissue, and maternal hair may provide insight into how stress-associated metabolic changes differ between maternal and fetal environments.

## 2. Materials and Methods

### 2.1. Study Participants

Nulliparous pregnant women (age ≥ 18, nonsmokers) were recruited during their third trimester at UCSF Mission Bay Hospital under UCSF IRB protocol #16-10957 [3]. Inclusion criteria were age ≥ 18, English or Spanish fluency, and consent to complete questionnaires and provide tissue samples. Exclusion criteria included any history of adrenal or endocrine disease, use of exogenous steroids (e.g., prednisone), smoking, or cognitive impairment. Perceived stress was measured using the 10-item Perceived Stress Scale (PSS-10) during the clinic visit, and sociodemographic data (age, education, income, etc.) were collected via a self-report questionnaire. Of 52 women who consented (Figure S2), 17 completed the study procedures (including the PSS questionnaire); final biospecimen availability was placenta (n = 12/17), maternal hair (n = 7/17), cord plasma (n = 6/17), and maternal plasma (n = 6/17). Gestational age and infant birth weight were recorded from medical records for all 17 participants [3].

### 2.2. Biospecimen Collection

At the third-trimester clinic visit, a small hair sample (~1–3 cm in length) was cut from the posterior scalp and stored in a foil-wrapped bag at room temperature. None of the women had recent hair treatments. At delivery, placentas were kept at 4 °C and collected within 2–12 h postpartum. Maternal peripheral blood and umbilical cord blood were drawn into EDTA tubes at delivery and processed to plasma within 12 h. All collected samples (plasma, tissue, hair) were aliquoted and stored at –80 °C until analysis. Placental tissue was sampled by punching five biopsies through the full thickness of the placenta, from the uterine to the fetal surface (Figure S3). This sampling pattern was applied consistently across all placentae to ensure representative coverage of different regions.

### 2.3. Metabolite Analysis

Metabolomic profiling included targeted quantification of steroid hormones and bile acids by ultra-performance liquid chromatography–tandem mass spectrometry (UPLC MS/MS) at UC Davis West Coast Metabolomic Center (WCMC). In brief, ~15 mg of placental tissue (or equivalent plasma/hair sample) was homogenized in an antioxidant solution (0.2 mg/mL BHT/EDTA in 1:1 methanol: water), centrifuged, and subjected to serial solvent washes. Extracts were lyophilized and reconstituted in 100 μL of a methanol/acetonitrile (50:50) solvent containing phenyl-hexadecanoic acid/urate (PHAU/CUDA) as internal standards (Supplementary Methods and Table S1). An injection volume of 5 µL was used, and normalized concentration was calculated as =((Sample Value (nM) × (Acc Mass × 10 × 10^−9^) × (Inj Vol × 10 × 10^−6^))/Unit Changer) × (Dil Factor (20)/mg Sample used)). Tritiated steroids were added to monitor HPLC retention and recovery, and deuterated steroid analogs were spiked into each sample to calibrate quantification. Concentrations were calculated from the peak area ratio to the corresponding deuterated internal standard and normalized to tissue weight (pg/mg) or plasma volume (ng/mL) as above and described before [3].

For bile acids, a parallel LC-MS/MS assay was used to quantify a targeted panel of conjugated bile acids. Analytes were detected by negative-ion MRM on the same mass spectrometer. Calibration curves were generated using authentic bile acid standards (e.g., cholic acid, chenodeoxycholic acid, glycochenodeoxycholic acid, ursodeoxycholic acid, etc.) (Supplementary Table S1), and concentrations in samples were calculated relative to internal standards of known quantity [20,21,22]. Matrix-specific LOD and LOQ values, detection frequency, and the number of quantified observations for every analyte and compartment are provided in Supplementary Table S2.

### 2.4. Statistical Analysis and Data Availability

Data was analyzed using Aseesa Stars 2.0 (https://www.aseesa.com/) and as described before [23]. Participants who did not contribute a biospecimen to a given biological compartment out of the total seventeen participants (N = 17) were excluded from the denominator and are not coded as zero; no values were imputed, and there were no missing values from any biological compartment for the bile acids or the steroids.

PSS was retained as a continuous variable for the primary analysis. Each of the 53 analytes was evaluated separately in maternal plasma, cord plasma, placenta, and maternal hair, giving 212 possible analyte-compartment pairs. Thirty-eight pairs met the 80% criterion. Quantitative association testing was restricted to pairs for which at least 80% of observations were at or above the supplied LOD. We used an LOD-based eligibility criterion because many physiologically relevant values fall in that range, and meeting the LOD-based criterion indicates reliable detection, not confirmed quantitative accuracy. Within eligible pairs, observations below the LOD were assigned LOD/sqrt(2) for rank-based analysis; original instrument values and censoring indicators were retained in the analysis dataset. When reporting ineligible pairs, including maternal-plasma UDCA and plasma/placental progestogens (above LOD in 50% (3/6 or 6/12, respectively) of participants), they were summarized using detection frequencies and were not assigned quantitative association estimates.

Kendall tau-b was the primary measure of association between PSS and metabolite concentration because it accommodates tied ranks and is appropriate for small samples. Spearman rho was calculated as a supplementary measure. Uncertainty was estimated using 10,000 participant-level paired bootstrap replicates. Two-sided permutation *p*-values were calculated exactly when complete enumeration was computationally feasible; otherwise, 100,000 Monte Carlo permutations with a fixed random seed and the (b + 1)/(m + 1) correction were used. Benjamini–Hochberg false-discovery-rate correction was applied across all 38 eligible PSS-analyte tests. Leave-one-participant-out analyses evaluated the influence of individual observations. The cohort-derived PSS division of ≤11 versus >11.1 was retained only as a secondary sensitivity analysis. Missing clinical covariates were not imputed. In addition to Aseesa Stars (V2.0), in-house Python 3.14.10 scripts were used for the analyses. Individual data files are provided as Supplementary Tables S3–S6; analytic samples may not be representative of the broader female population.

## 3. Results

### 3.1. Centering vs. Individual Care: PSS and Sociodemographic Scores Were Not Different

Because Centering Pregnancy has previously been associated with reduced stress and improved psychological support, and this study was originally designed to compare perceived stress between women receiving Centering versus Individual prenatal care, we first evaluated whether perceived stress levels differed between prenatal care models in this cohort. Determining whether prenatal care type influenced PSS scores was important to assess whether subsequent metabolomic analyses should be stratified by care model as a secondary analysis or by perceived stress alone. A continuous PSS analysis was retained for primary analysis. Participant demographic and clinical characteristics are summarized in Table 1. The cohort included nulliparous women receiving either Centering Pregnancy group prenatal care or traditional individual prenatal care at UCSF Mission Bay Hospital. Clinical variables collected from medical records included gestational age at delivery, maternal body mass index (BMI), infant birth weight, and delivery mode.

PSS scores ranged from 1 to 23 among 17 participants, with a median of 11 and an interquartile range of 9–14. Median PSS was 12.5 among participants receiving Centering care (n = 4) and 11.0 among those receiving individual care (n = 13). The exploratory exact label-permutation Mann–Whitney comparison did not provide evidence of a difference between care models (U = 32, two-sided *p* = 0.536). Given the small Centering subgroup, this finding does not establish equivalence or exclude care model as a potential confounder. The descriptive division between scores 11.0 and 11.1 produced 9 participants with PSS ≤ 11.0 and 8 with PSS ≥ 11.1; continuous PSS was used in the primary analyses (Figure 1).

Median perceived stress and sociodemographic (SES) scores did not significantly differ between participants enrolled in Centering Pregnancy and those receiving individual prenatal care (Centering: median PSS 12.5 and SES 33.5; Individual care: median PSS 11 and SES 34; *p* = n.s.) (Figure 1C,D). Median maternal BMI (26.95 vs. 27) and age (35.5 vs. 33 years) also did not differ between Centering and Individual care participants (Figure S4A,B). Due to the small number of participants enrolled in the Centering Pregnancy cohort (n = 4), this comparison was considered exploratory and insufficiently powered for definitive statistical interpretation.

Because perceived stress scores were comparable between prenatal care models, PSS was retained as a continuous variable for the primary analysis. As a secondary, exploratory sensitivity analysis only, participants were also divided using a cohort-derived descriptive threshold into Low-PSS (≤11.0; n = 9) and High-PSS (≥11.1; n = 8) groups, as shown in Figure 1E,F. This division was not used to establish significance and does not represent a validated clinical cutoff. Sample sizes differed across individual metabolite analyses because not all participants contributed every biospecimen type.

Delivery outcomes in this cohort were generally favorable. Gestational age at delivery ranged from 37 + 0 to 41 + 6 weeks; there were no preterm deliveries. Thirteen of 17 participants (76.5%) delivered vaginally (NSVD), 3 (17.6%) by cesarean section (LTCS), and one (5.9%) by vacuum-assisted vaginal delivery. Birth weight ranged from 2360 to 4260 g (mean 3304 g), within the normal range for all infants. Five-minute Apgar scores were 8 or 9 for all 17 infants, and no infant was admitted to the NICU. Despite these reassuring outcomes, 13 of 17 participants (76.5%) had at least one documented clinical complication or comorbidity, most commonly gestational diabetes (8/17, 47.1%), with additional cases of gestational hypertension, chorioamnionitis, polycystic ovary syndrome, possible Zika exposure, and prior cesarean delivery. PSS scores ranged from 1 to 23 (median 11.0; IQR: 4.99–17.00), socioeconomic scores ranged from 25 to 55 (median 34; IQR: 26.17–41.83), maternal BMI ranged from 22.3 to 52.2 (median 27.0; IQR: 19.81–34.19), maternal age ranged from 29 to 40 years (median 34 years; IQR: 30.36–37.63), and infant birth weight ranged from 2360 g to 4260 g (median 3390 g; IQR: 2866.49–3913.50; Figure S4). This combination of a substantial comorbidity burden and wide variability in perceived and/or socioeconomic stressors, alongside uniformly favorable delivery outcomes, provides the clinical backdrop for the metabolic findings described below.

### 3.2. Overview of Metabolites Across Four Biological Compartments

Before evaluating stress-associated metabolomic differences, it was necessary to determine which steroid and bile acid metabolites were reliably detected across maternal and fetal samples. This study characterized steroid and bile acid metabolites across four maternal–fetal compartments: maternal plasma, cord plasma, placental tissue, and maternal hair. Detection frequency varied markedly across analytes and biological compartments, with only 21 metabolites meeting the ≥80% prerequired threshold. Of 212 possible analyte-compartment pairs, 38 metabolites met the predefined requirement of at least 80% observations at or above the LOD and were tested for association with PSS and sociodemographic scores using Kendall’s tau b, with permutation *p*-values, bootstrap confidence intervals, and Benjamini–Hochberg correction for multiple comparisons. Fourteen pairs were eligible in cord plasma, 12 in maternal plasma, 11 in placenta, and one in maternal hair (Table 2 and Table 3 and Figure 2 and Figure 3).

In the steroid metabolite panel, 33.33% (10/30) of steroid metabolites were present above the limit of detection (LOD) levels in at least one biospecimen in ≥80.0% of observations (Table 2). Cord plasma contained 26.66% (8/30) and maternal plasma contained 13.33% (4/30) detectable steroid metabolites, whereas placental tissue contained 23.33% (7/30) detectable steroid metabolites. In contrast, no steroid metabolites (0/30) were above the LOD in maternal hair samples meeting the ≥80.0% threshold.

In the bile acid panel, 43.47% (10/23) of metabolites were above the LOD threshold in at least one compartment at ≥80.0% observations (Table 3). Maternal plasma demonstrated the broadest bile acid detectability, with 34.78% (8/23) of bile acids above LOD, followed by cord plasma with 26.08% (6/23), and placental tissue with 17.39% (4/23) detectable bile acids each. Maternal hair demonstrated lower analyte recovery, with only 4.34% (1/23) of bile acids above LOD. Overall, placental tissue and maternal plasma exhibited the greatest metabolite detectability across both steroid and bile acid panels, whereas maternal hair showed the least number of metabolites with ≥50% observations.

### 3.3. PSS- and Sociodemographic Score (SES)-Associated Metabolite Changes

To identify metabolites potentially associated with maternal perceived stress, we first ascertained Kendall’s tau-b coefficients between continuous perceived stress and sociodemographic scores and analyte concentrations. None of the 38 quantitatively eligible ≥80% threshold analyte-compartment associations survived Benjamini–Hochberg FDR correction. The smallest primary permutation *p*-values were observed for cord-plasma estradiol (n = 6, Kendall tau-b= −0.733, 95% bootstrap CI −1.000 to −0.111, *p* = 0.0556, q = 1.00), maternal-plasma cortisol (n = 6, tau-b = −0.733, 95% CI −1.000 to 0.000, *p* = 0.0556, q = 1.00), placental estrone (n = 12, tau-b = −0.382, 95% CI −0.821 to 0.085, Monte Carlo permutation *p* = 0.0976, q = 1.00) and cord-plasma GCDCA (n = 6, tau-b = −0.600, 95% CI −1.000 to 0.231, *p* = 0.136, q = 1.00). The data are depicted via forest plots in Figure 3.

In secondary exploratory analysis, we compared metabolite abundance between High-PSS and Low-PSS pregnancies across all maternal and fetal compartments. To evaluate global metabolomic differences associated with maternal perceived stress, volcano plot analysis was performed to compare the High-PSS and Low-PSS groups across all steroid and bile acid metabolites regardless of LOD value cutoff (Figure 4). Four steroid and two bile acid metabolites within the placental, maternal, and cord plasma compartments were nominally associated with high PSS (uncorrected *p* < 0.05; Figure 4).

### 3.4. Bile Acid Profiles and PSS-Associated Changes

Given the emerging role of bile acids during pregnancy, in secondary analysis we evaluated whether maternal perceived stress was associated with alterations in bile acid profiles across maternal and fetal compartments. Bile acid concentrations and metabolite composition differed substantially across maternal and fetal compartments, suggesting compartment-specific regulation of bile acid metabolism during pregnancy. Two bile acids demonstrated nominal PSS-associated differences consistent with the volcano plot analysis: glycochenodeoxycholic acid (GCDCA) in cord plasma and ursodeoxycholic acid (UDCA) in maternal plasma. Cord plasma GCDCA concentrations and maternal plasma UDCA concentrations were reduced in participants with higher perceived stress scores (Figure 5, uncorrected *p* < 0.05; did not survive multiple-testing correction).

### 3.5. Estrone (E1), a Lesser-Known Player

The three principal estrogens during pregnancy are E1, E2, and E3. Because estrogens are often evaluated collectively despite possessing distinct biological functions, we next examined whether specific estrogen metabolites demonstrated differential associations with maternal perceived stress. In secondary analysis, a nominal reduction in placental E1 concentrations among participants with higher perceived stress scores (uncorrected *p* < 0.05; did not survive multiple-testing correction) (Figure 6) was noted. In contrast, neither E2 nor E3 demonstrated significant PSS-associated differences in any maternal or fetal compartment. We have previously shown that cord estradiol associates negatively (r = –0.943, *p* = 0.017) with PSS as a continuous variable in this cohort, whereas placental E2 does not [3].

### 3.6. Gestational Diabetes Mellitus Status Does Not Alter Postpartum Steroid and Bile Acid Metabolite Levels

As gestational diabetes mellitus status is known to adversely affect maternal and fetal outcomes as well as alter steroid and bile acid metabolites to regulate insulin levels, we next ascertained the status of these parameters. Neither PSS nor SES differed significantly between women who developed GDM and those who did not (Figure 7A,B). Median maternal age was 36.5 years in women with GDM versus 33 years in women without GDM (Figure 7C). We ascertained the distribution of GDM, infant sex, and BMI across PSS groups. None differed between Low- and High-PSS groups (GDM 3/9 vs. 5/8, *p* = 0.35; female infants 5/9 vs. 6/8, *p* = 0.62; median BMI 26.9 vs. 27.4 kg/m^2^, *p* = 0.42), and PSS was not associated with BMI (Kendall τ = 0.20, *p* = 0.26). Infants’ sex (Figure 7D) and birth weight (Figure 7E) did not differ with PSS x GDM status, nor was there any interaction effect for birth weight (*p* = 0.654; F-statistic = 0.785, two-way ANOVA). Maternal BMI likewise did not differ by GDM status alone or across PSS x GDM subgroups (Figure 7F). No bile acid metabolite differed by GDM status (Figure 7G). In secondary exploratory analysis, median concentrations of two steroid metabolites: dihydroprogesterone (DHP, *p* = 0.017; n = 12) and pregnenolone (PELN, *p* = 0.024; n = 12) were lower in women who developed GDM versus those who did not (Figure 7H). Levels of 20-hydroxyprogesterone (20OH-PROG) were nominally lower in maternal plasma (n = 6; Figure 7H). Most of these metabolites met the ≥50%, not ≥80%, LOD threshold and are reported because of their physiological significance in pregnancy. We repeated the primary analysis (i) excluding participants with GDM, (ii) stratified by GDM, (iii) stratified by infant sex, and (iv) excluding the participant with BMI 52.2 (Figure 7I). The direction of the strongest associations, cord-plasma estradiol and maternal-plasma cortisol, was preserved across analyses, and no association reached FDR significance in any analysis. The inverse association of placental estrone with PSS strengthened when the participant with BMI 52.2 was excluded but was attenuated after excluding participants with GDM.

### 3.7. Hair Is Not a Reliable Compartment for Steroid and Bile Acid Metabolite Levels

Because maternal hair has been proposed as a noninvasive biomarker of long-term hormonal exposure [24], we evaluated whether metabolite concentrations detected in hair reflected those observed in maternal plasma. Assessing agreement between hair and plasma metabolite profiles is important for evaluating the feasibility of hair-based metabolomics in pregnancy research. In this study, the majority of steroid and bile acid metabolites detectable in plasma or placental tissue were below the limit of detection in maternal hair samples. Of the 23 bile acid metabolites, only one bile acid, lithocholic acid (LCA), was present above LOD levels in hair (Figure 2, Table 3), and only one (1/30) steroid metabolite, progesterone, was previously reported to be above LOD [3].

## 4. Discussion

### 4.1. The Placenta: Buffering Capacity Hypothesis

Maternal psychological stress during pregnancy and gestational diabetes are associated with adverse maternal and neonatal outcomes, including cesarean delivery, preterm birth, low birth weight, and altered neurodevelopmental trajectories in offspring [16]. Previous metabolomic studies have demonstrated that psychological distress during pregnancy is associated with measurable alterations in lipid, amino acid, and inflammatory metabolic pathways [3,7]. Because the placenta functions as the primary endocrine and metabolic interface between mother and fetus, understanding how maternal stress alters placental metabolism is essential for identifying biological pathways associated with these outcomes. Gestational diabetes was a predominant co-occurrence, with 41.05% (8/17 women) exhibiting this endocrine disorder. Maternal age and BMI showed similar median values regardless of how the cohort was stratified, whether by PSS alone (Supplementary Figure S5), GDM alone (Figure 7C,F inset), or their combination (Figure 7F), arguing against either variable as a major independent driver of the association reported here. In the present study, most steroid and bile acid metabolites remained relatively stable and displayed no significant association with PSS, sociodemographic scores, or gestational diabetes, in primary or secondary analyses. A non-significant result in this small, exploratory cohort does not itself demonstrate stability, equivalence, or homeostasis; it is, however, consistent with the possibility that healthy term placentas may effectively maintain endocrine and metabolic homeostasis despite maternal psychological stress exposure and different sociodemographic status. These findings are in agreement with prior work proposing that the placenta serves as a protective biochemical buffer that regulates fetal exposure to maternal stress-associated hormones and metabolites [3].

Our earlier work in this same cohort first proposed that the human placenta buffers the fetus from many effects of maternal perceived stress [3], and the present findings, drawn from a different set of metabolites in the same participants, reinforce that conclusion rather than standing apart from it. The placenta has no real analog elsewhere in human physiology: it is built anew with each pregnancy, discarded within minutes of delivery, and yet for nine months carries out the combined work of a maternal–fetal lung, gut, kidney, and endocrine gland. Its buffering role is also not specific to psychological stress. The same transport and metabolic machinery that governs fetal hormone exposure is also responsible for limiting fetal exposure to circulating xenobiotics and environmental toxicants, and disruption of placental transport and metabolic pathways has been implicated in adverse outcomes following maternal exposure to air pollution and other chemical stressors [25]. Read alongside our prior report, the present results support a broader view in which a healthy placenta acts as a general-purpose buffer against maternal stressors of different kinds, psychological and chemical alike, and in which the integrity of that buffering capacity, rather than the stressor itself, may be what most directly shapes fetal outcomes.

Placental E1, cord plasma GCDCA, and maternal plasma UDCA showed nominal PSS-associated differences that did not survive multiple-testing correction. If replicated in an adequately powered study, such a pattern would be consistent with the notion that placental buffering may be perturbed selectively across specific metabolic pathways after exposure to certain stressors/insults during pregnancy. Rather than broad dysregulation of steroidogenesis or bile acid metabolism, maternal perceived stress appeared, in this small sample, to be nominally associated with a limited subset of metabolites. One possible explanation is compensatory placental regulatory mechanisms that preserve overall homeostasis while allowing subtle alterations in stress-sensitive pathways. Prior studies in both humans and animal models suggest that relative metabolite balance may be more biologically important than absolute metabolite concentrations during pregnancy [26]. Our exploratory findings are consistent with this concept, though they do not establish it, and are compatible with the possibility that healthy pregnancies may tolerate moderate maternal stress exposure while maintaining overall fetal endocrine stability.

While placental cortisol was below the LOD, placental cortisone was clearly detected (median 371.2 pg/mg). This pattern is consistent with placental 11β-HSD2 activity, the enzyme that inactivates cortisol to cortisone and that we describe as part of the maternal–fetal stress response system. We did not perform additional analysis to confirm this mechanism directly, but the observation fits the broader framework and offers one concrete, testable example of the placental buffering in this study set out to characterize. This cohort’s clinical profile lends credence to this notion. Despite a substantial comorbidity burden (76.5% of participants had at least one documented complication, most often gestational diabetes), a wide range of maternal BMI, sociodemographic factors, a substantial proportion of older, first-time mothers (41.1% ≥ 35 years (7/17)), and perceived stress scores, delivery outcomes were uniformly favorable: no preterm births, appropriate birth weights, reassuring 5 min Apgar scores in every infant, and no NICU admissions. We did not test this pattern statistically, and a cohort of 17 cannot establish that placental buffering, rather than chance, small effect size, or factors outside the data set, explains it. Still, the co-occurrence of substantial maternal physiological and psychological variability with consistently good neonatal outcomes is the kind of pattern a buffering placenta would be expected to produce, and it motivates the more specific, metabolite-level question this study was designed to address.

The GM findings provide a second, mechanistically independent line of support for our hypothesis. Unlike perceived stress, GDM is a diagnosed clinical condition rather than a self-reported measure, and nearly half of this cohort developed it. PSS and sociodemographic scores did not differ between women who did and did not develop GDM. Importantly, the primary PSS metabolite associations were materially unchanged whether GDM cases were included, excluded, or used to stratify the analysis (Figure 7I). A modest attenuation of the placental estrone association after excluding GDM cases and a few individually nominal progestogens differences by GDM status were noted. That metabolite levels remain largely stable across 2 mechanistically distinct stressors, one psychological and self-reported, the other physiological and clinically diagnosed, is consistent with our broad placental buffering capacity hypothesis across stressor types regardless of qualitative or quantitative measurement of the given stressor.

These findings may also help explain why adverse birth outcomes are not uniformly observed in all pregnancies affected by maternal stress. Even in the presence of measurable maternal psychological stress, placental metabolic regulation may partially protect the fetus from large-scale endocrine disruption. At the same time, selective alterations in metabolites such as E1, GCDCA, and UDCA may represent early biochemical indicators of stress-associated physiological adaptation that could become more pronounced in pregnancies complicated by placental dysfunction, preterm birth, or metabolic disease. Distinct metabolomic alterations have previously been reported in pregnancies complicated by preeclampsia and preterm delivery, supporting the possibility that metabolic dysregulation becomes more evident in pathologic pregnancies [5]. Together, these findings suggest that maternal perceived stress may influence highly specific metabolic pathways rather than causing broad endocrine disruption during healthy term pregnancy.

### 4.2. Why E1 and Not E2/E3?

One of the most unexpected findings in this study was the selective reduction in placental E1 in participants with higher perceived stress scores. Our earlier report, using continuous PSS correlations, found cord plasma estradiol negatively associated with PSS and hair progesterone positively associated with PSS [3]. The present study’s different analytic framework and broader hair panel mean these single-analyte findings are not directly comparable to the current results and should not be interpreted as contradicting them. Most prior studies investigating estrogen signaling during pregnancy have focused primarily on E2 or have grouped estrogens together rather than examining individual estrogen metabolites separately. As a result, the possibility that maternal stress may selectively alter E1 rather than E2 has received little attention.

Although E2 is generally considered the dominant biologically active circulating estrogen in nonpregnant women, E1 possesses distinct metabolic and physiological roles. During pregnancy, placental steroidogenesis involves dynamic interconversion between E1 and E2 through the activity of hydroxysteroid 17- β dehydrogenase enzymes, including HSD17β1 [27]. Maternal stress-associated alterations in placental enzyme activity could therefore shift the balance between E1 and E2 production without necessarily altering total estrogen abundance. This possibility is biologically plausible given that glucocorticoid signaling has been shown to influence placental steroidogenic pathways and endocrine regulation.

Selective depletion of E1 may have important implications for maternal and fetal physiology. Estrogens contribute to placental vascular function, cervical remodeling, fetal organ maturation, and preparation for labor [4]. If maternal stress preferentially alters E1 metabolism, these changes could affect downstream estrogen-sensitive processes in ways that would not be detected when measuring only E2 or total estrogen levels.

These findings also highlight the importance of modern mass spectrometry approaches capable of measuring steroid metabolites individually rather than grouping hormones together. Earlier immunoassay-based studies often lacked the specificity required to distinguish compartment-specific estrogen alterations [28]. Our results suggest that future pregnancy metabolomics studies should evaluate E1, E2, and E3 separately, particularly in the context of maternal stress and placental function.

### 4.3. Bile Acids in Pregnancy, a Less Explored Territory

In addition to steroid hormones, this study characterized bile acid metabolites across maternal plasma, placental tissue, cord plasma, and maternal hair in relation to maternal perceived stress. To our knowledge, this represents one of the first studies to simultaneously evaluate bile acid profiles across multiple maternal–fetal compartments in the context of maternal psychological stress. Bile acids are increasingly recognized not only as regulators of lipid digestion but also as signaling molecules involved in inflammation, glucose metabolism, liver function, and endocrine regulation. Bile acids exert many of these regulatory effects through signaling receptors, including FXR and TGR5, which influence glucose homeostasis, inflammatory signaling, and lipid metabolism [12]. Despite growing interest in bile acid metabolism during pregnancy, little is known about how maternal stress may influence bile acid homeostasis across maternal and fetal tissues.

Two bile acids showed nominal, uncorrected PSS-associated changes in this cohort: glycochenodeoxycholic acid (GCDCA) in cord plasma and ursodeoxycholic acid (UDCA) in maternal plasma. GCDCA is a conjugated primary bile acid involved in hepatic cholesterol metabolism and bile acid transport. Reduced cord plasma GCDCA levels in high-PSS pregnancies may reflect altered maternal–fetal bile acid transfer or stress-associated changes in placental bile acid regulation [29]. This is consistent with evidence from a pregnant swine showing that placental bile acid handling normally limits transfer of maternal bile acids into the fetal compartment, implying that fetal bile acid levels depend as much on placental transport capacity as on maternal supply [30]. One plausible route by which psychological stress could alter that capacity is inflammatory signaling: in a pregnant rodent model, acute systemic inflammation downregulated placental expression of several bile acid and drug transporters, altering maternal-to-fetal disposition of endogenous compounds [31,32]. If a similar inflammatory pathway operates in chronically stressed human pregnancies, it will offer a testable mechanistic link between perceived stress and the reduced cord plasma GCDCA observed here, though this remains speculative until directly examined. Because bile acids can cross the placental interface, alterations in placental transport mechanisms may contribute to compartment-specific bile acid accumulation or depletion. Altered fetal bile acid exposure may be biologically important because bile acids function not only in hepatic metabolism but also as signaling molecules involved in inflammatory regulation, glucose homeostasis, and cellular stress responses. Comprehensive bile acid profiling studies have shown that bile acid dysregulation in pregnancy is associated with widespread metabolic and endocrine pathway alterations [14]. Consequently, even modest stress-associated changes in bile acid composition could influence fetal metabolic programming or placental inflammatory signaling.

The observed reduction in maternal plasma UDCA is particularly interesting, given the established anti-inflammatory and hepatoprotective properties of UDCA. Beyond its role in bile flow, UDCA modulates inflammatory and immune signaling through several distinct mechanisms, including antioxidant, anti-apoptotic, endoplasmic-reticulum stress-relieving, and direct immunomodulatory effects, which together make it a plausible buffer against stress-associated inflammatory changes rather than a purely hepatic actor [33]. Clinically, UDCA is used as a therapeutic treatment for intrahepatic cholestasis of pregnancy, where it helps reduce maternal bile acid accumulation and improve fetal outcomes [13,34], including reduction in preterm and stillbirths in a meta-analysis [35], whereas data from the placebo-controlled PITCHES trial did not reduce adverse perinatal outcomes [36]; hence, the recommendation remains equivocal. Lower UDCA concentrations in participants with higher perceived stress scores may therefore indicate stress-associated disruption of bile acid regulatory pathways. Psychological stress has previously been linked to inflammatory and metabolic dysregulation, including altered liver-associated signaling pathways, making stress–bile acid interaction biologically plausible. Metabolomic analyses of pregnant individuals with depression have identified disruptions in multiple metabolic and inflammatory pathways associated with psychological stress exposure [3,7].

The identification of stress-associated bile acid alterations across maternal and fetal compartments provides preliminary evidence that maternal psychological stress may influence pathways beyond classical glucocorticoid signaling.

### 4.4. Hair: A Less Explored Biological Compartment

Maternal hair was included as an exploratory noninvasive matrix for cumulative exposure during pregnancy [24]. In this cohort, however, hair and plasma showed a basic mismatch rather than a simple sensitivity gap (Section 3.6): metabolites abundant in plasma were not consistently detected in hair, and the direction of change did not reliably match. This may reflect biological differences in metabolite incorporation, degradation, and keratin binding, as well as the different time windows the two matrices represent [37]. We therefore treat the hair data as exploratory only and do not base any conclusions on them; analyte-specific validation would be required before hair could substitute for plasma-based metabolite assessment. Plasma metabolites reflect relatively acute physiological states, whereas hair may capture longer-term cumulative exposure. As a result, these tissues may provide fundamentally different biological information rather than interchangeable measures of the same process. Although hair-based metabolomics remains promising as a noninvasive biomarker strategy, our results suggest that analyte-specific validation is necessary before hair measurements can reliably substitute for plasma-based metabolite assessment during pregnancy.

### 4.5. Limitations

Several limitations should be considered when interpreting the findings of this study, and most stem from a constraint that is characteristic of this area of research rather than specific to our design: simultaneously collecting matched maternal plasma, hair, placental tissue, and cord plasma from term pregnancies is logistically demanding, and cohorts with all four compartments represented remain uncommon in the literature. Participant recruitment and enrollment for this cohort were described in our earlier publication [3], including the 52 women who consented and the 17 who completed study procedures (Figure S2); no additional participants were recruited for the present manuscript. We cannot rule out that the final analytic sample differs systematically from the broader clinic population, and we note this as a limitation. The overall cohort size was small, and biospecimen availability differed across compartments, resulting in unequal subgroup sizes for several analyses. This limited statistical power and reduced the ability to detect smaller stress-associated metabolite differences. We view this as a reason for caution in how far we extend biological interpretation of any single effect size, not as a reason to set the findings aside. Second, this study used a cross-sectional design with sample collection occurring at delivery, preventing longitudinal assessment of changes in perceived stress and metabolite dynamics throughout gestation. Maternal stress exposure likely fluctuates during pregnancy, and a single third-trimester PSS measurement may not fully capture cumulative psychological stress burden.

LOD and LOQ are strictly technical performance metrics of the instrument being used and do not reflect whether that concentration has a biologically relevant effect in vivo. An additional limitation is the lack of well-established reference ranges for postpartum placental E1 concentrations in healthy pregnancies, which also remain very low, like estradiol. Although placental E1 was nominally lower in participants with higher perceived stress scores, limited prior literature characterizing compartment-specific postpartum E1 dynamics makes interpretation of the biological directionality uncertain. Consequently, it remains unclear whether reduced placental E1 reflects altered placental synthesis, increased fetal transfer, compensatory metabolism, or other stress-associated regulatory mechanisms. Larger studies establishing normative compartment-specific estrogen profiles across pregnancy and delivery are needed to better contextualize these findings.

These constraints call for restraint in how far we extend biological interpretation, but they are not, in our view, a reason to set the findings aside. Given how rarely all four maternal-fetal compartments are sampled in the same cohort, even a modest, well-characterized signal has value in pointing toward where larger studies should look. This study provides a novel multi-compartment characterization of steroid and bile acid metabolites in relation to maternal perceived stress and identifies three candidate metabolites, placental E1, cord plasma GCDCA, and maternal plasma UDCA, that warrant targeted follow-up in larger, longitudinal cohorts.

## 5. Conclusions

This study provides the first integrated characterization of steroid and bile acid metabolites across maternal plasma, placental tissue, cord plasma, and maternal hair in relation to maternal perceived stress during term pregnancy. In the present study, most steroid and bile acid metabolites remained relatively stable and displayed no significant association with PSS, sociodemographic scores, or gestational diabetes, in primary or secondary analyses. In the secondary analysis, nominal, uncorrected differences in specific steroid and bile acid metabolites in placental tissue, cord, and maternal plasma are consistent with, though do not establish, the possibility that certain maternal stress selectively influences specific endocrine and metabolic pathways despite overall preservation of placental buffering capacity. As a broader point, the reported LOD/LOQ concentration units (ng/mL, pg/mg) reflect analytical (instrument limitation) rather than any established physiologically relevant threshold; it is not currently known what minimum local concentration is required at the cellular level to produce a given biological effect (e.g., receptor binding or transcriptional activation), and this uncertainty applies to cross-compartment comparisons generally, independent of the unit-harmonization issue for the four biological compartments. Together with our earlier report in this same cohort [3], these results reinforce a consistent picture: the human placenta is a uniquely capable buffering organ, one built anew each pregnancy to moderate fetal exposure not only to maternal psychological stress but, as the broader chemical-exposure literature suggests, to a range of environmental and toxicant stressors as well [25]. Placental health, in this framework, is not simply a marker of fetal growth but an active determinant of how much of the mother’s internal and external environment reaches the fetus at all. These findings support the concept that the placenta functions as an active metabolic regulator during pregnancy and identify potential metabolites that require further investigation. Larger longitudinal studies are needed to determine whether these metabolite alterations contribute to adverse pregnancy outcomes.

## Figures and Tables

**Figure 1:**
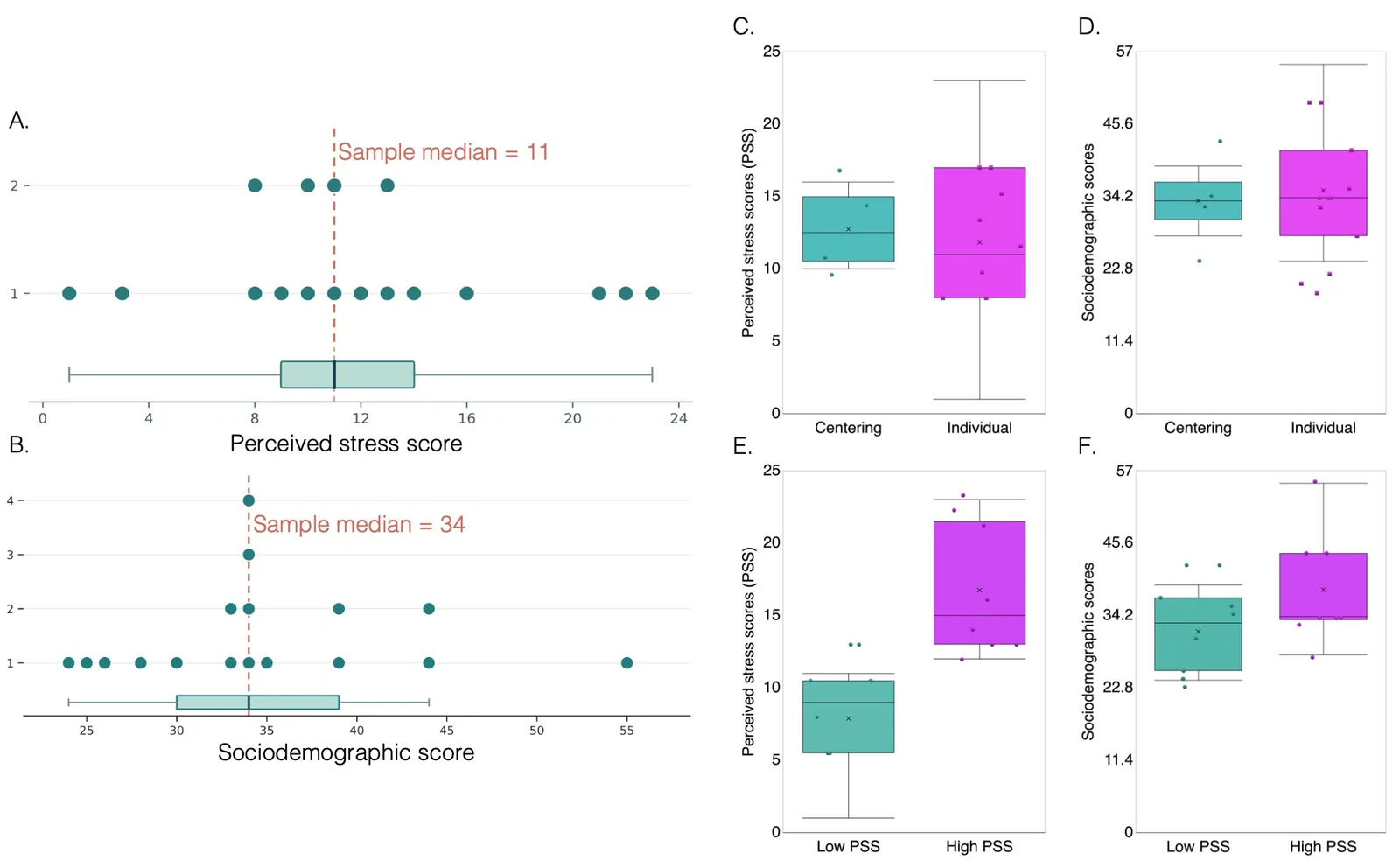
Perceived stress and sociodemographic scores in the cohort shown as continuous distributions and as discrete comparison groups. (**Left**) PSS score (**A**) and sociodemographic score (**B**) for each participant (n = 17) as continuous variables, plotted as the dot strip within the accompanying box-and-whisker plot. Points are stacked vertically only to reduce overlap between participants with identical scores. The dashed line marks the median and prenatal care model. The median PSS score was 11, the interquartile range (IQR) was 9–14, and the observed range was 1–23. The dashed vertical line at 11.5 denotes the cohort-specific descriptive division between low and high PSS; the median lines were not used to dichotomize participants or define inferential groups. (**Right**) The same 2 scores are shown as discrete comparisons: by prenatal care type (Centering, n = 4; Individual, n = 13; (**C**,**D**)) and by a cohort-derived, descriptive PSS threshold (Low-PSS ≤ 11.0, n = 9; High-PSS ≥ 11.1, n = 8; (**E**,**F**)). Boxes show the interquartile range with the median as the horizontal line and the mean (×); whiskers show the full range; points show individual participants’ values. PSS and sociodemographic scores did not differ significantly by care type. Because the Low-PSS/High-PSS grouping is defined directly from the PSS score, the corresponding PSS comparison bottom left is tautological by construction and is shown for descriptive orientation only; sociodemographic scores did not differ significantly between these same groups. The exploratory one-sided exact label-permutation Mann–Whitney comparison gave U = 32 and *p* = 0.536. *** means *p* < 0.001.

**Figure 2:**
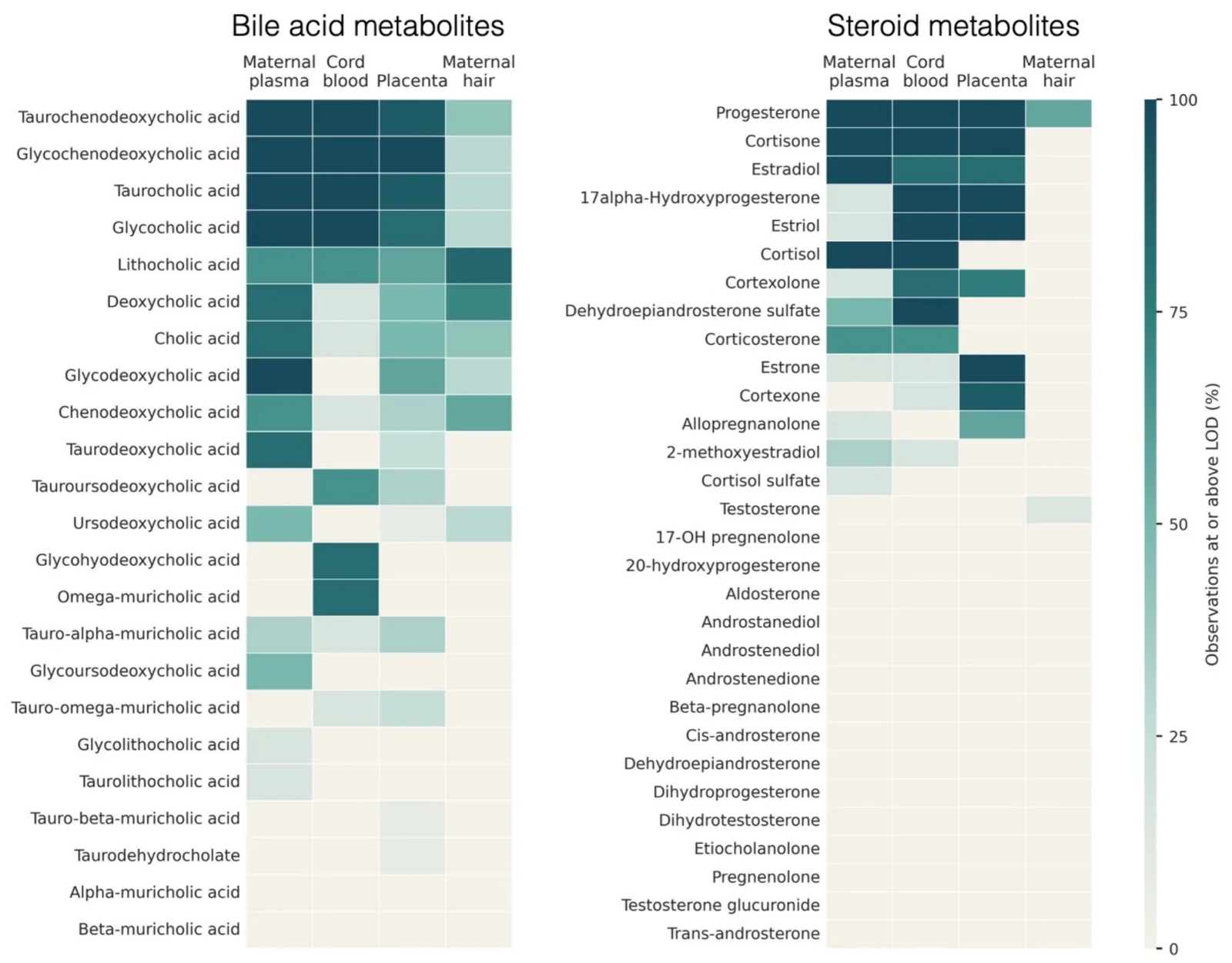
Detection frequency of targeted bile acid and steroid analytes across maternal plasma, cord plasma, placenta, and maternal hair. The left heatmap presents bile-acid analytes, and the right heatmap presents steroid analytes. Each cell represents the percentage of available observations at or above the supplied limit of detection (LOD) in maternal plasma (n = 6), cord plasma (n = 6), placenta (n = 12), and maternal hair (n = 7). Darker colors indicate a greater percentage of observations at or above the LOD. The heatmap reports analytical detectability and must not be interpreted as a comparison of metabolite concentrations or relative abundance across compartments. An analyte–compartment pair was considered quantitatively eligible when at least 80% of observations were at or above the LOD.

**Figure 3:**
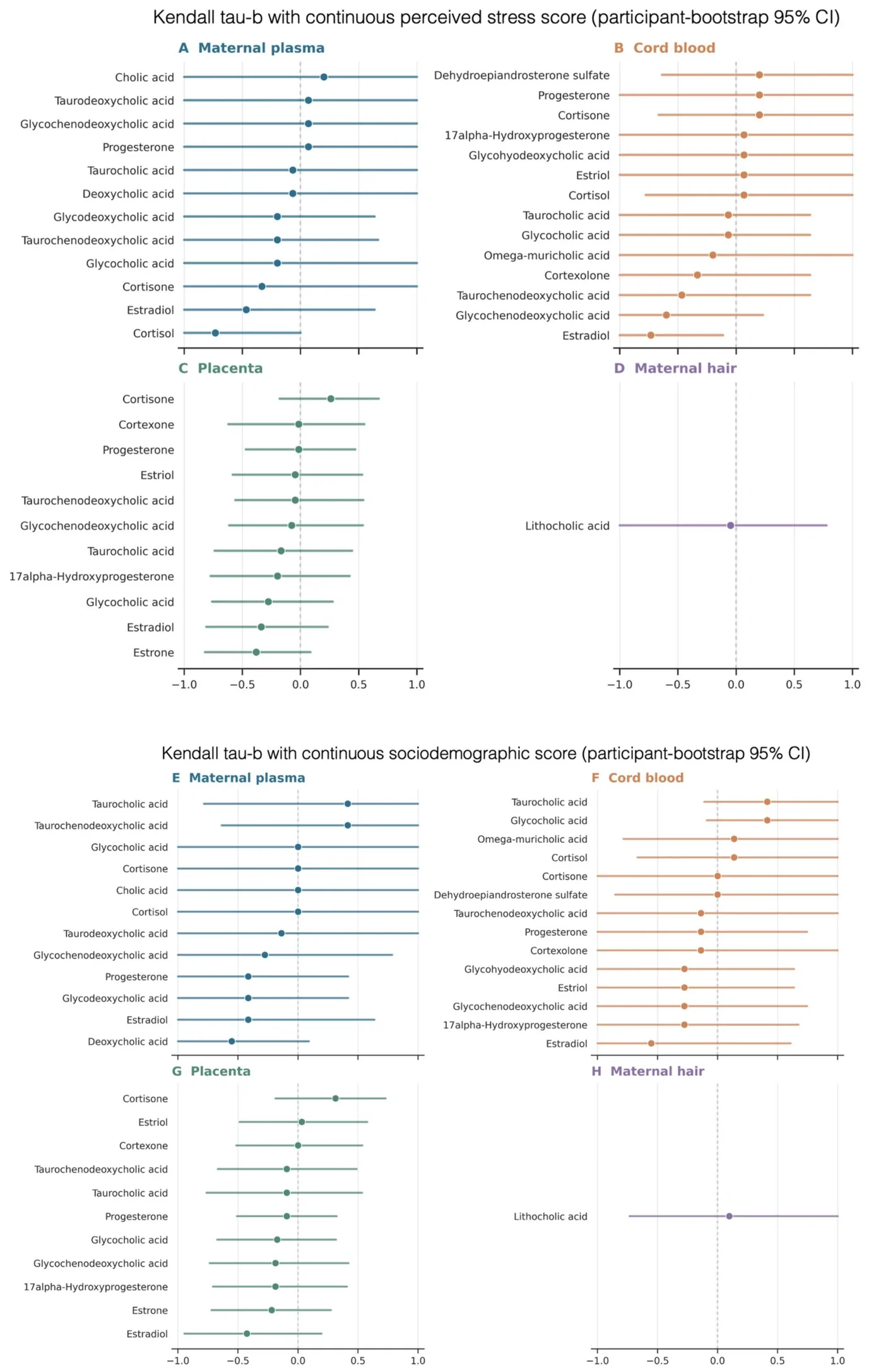
PSS: Panels show associations in (**A**) maternal plasma, (**B**) cord blood, (**C**) placenta, and (**D**) maternal hair. Points represent Kendall’s tau-b coefficients between continuous PSS-10 scores and analyte concentrations, and horizontal lines represent participant-level bootstrap 95% confidence intervals. The vertical dashed line at tau-b = 0 denotes no ordinal association. The figure includes all 38 analyte–compartment pairs meeting the prespecified quantitative eligibility criterion of at least 80% of observations at or above the limit of detection (LOD): 12 pairs in maternal plasma, 14 in cord blood, 11 in placenta, and one in maternal hair. Two-sided permutation *p*-values were adjusted across the complete 38-test PSS family using the Benjamini–Hochberg false-discovery-rate procedure. SES: Panels show associations in (**E**) maternal plasma, (**F**) cord blood, (**G**) placenta, and (**H**) maternal hair. Points represent Kendall’s tau-b coefficients between continuous SESs and analyte concentrations, and horizontal lines represent participant-level bootstrap 95% confidence intervals. The vertical dashed line at tau-b = 0 denotes no ordinal association. The same 38 analyte–compartment pairs meeting the prespecified criterion of at least 80% of observations at or above the LOD were examined. Two-sided permutation *p*-values were adjusted across the complete 38-test SES family using the Benjamini–Hochberg procedure.

**Figure 4:**
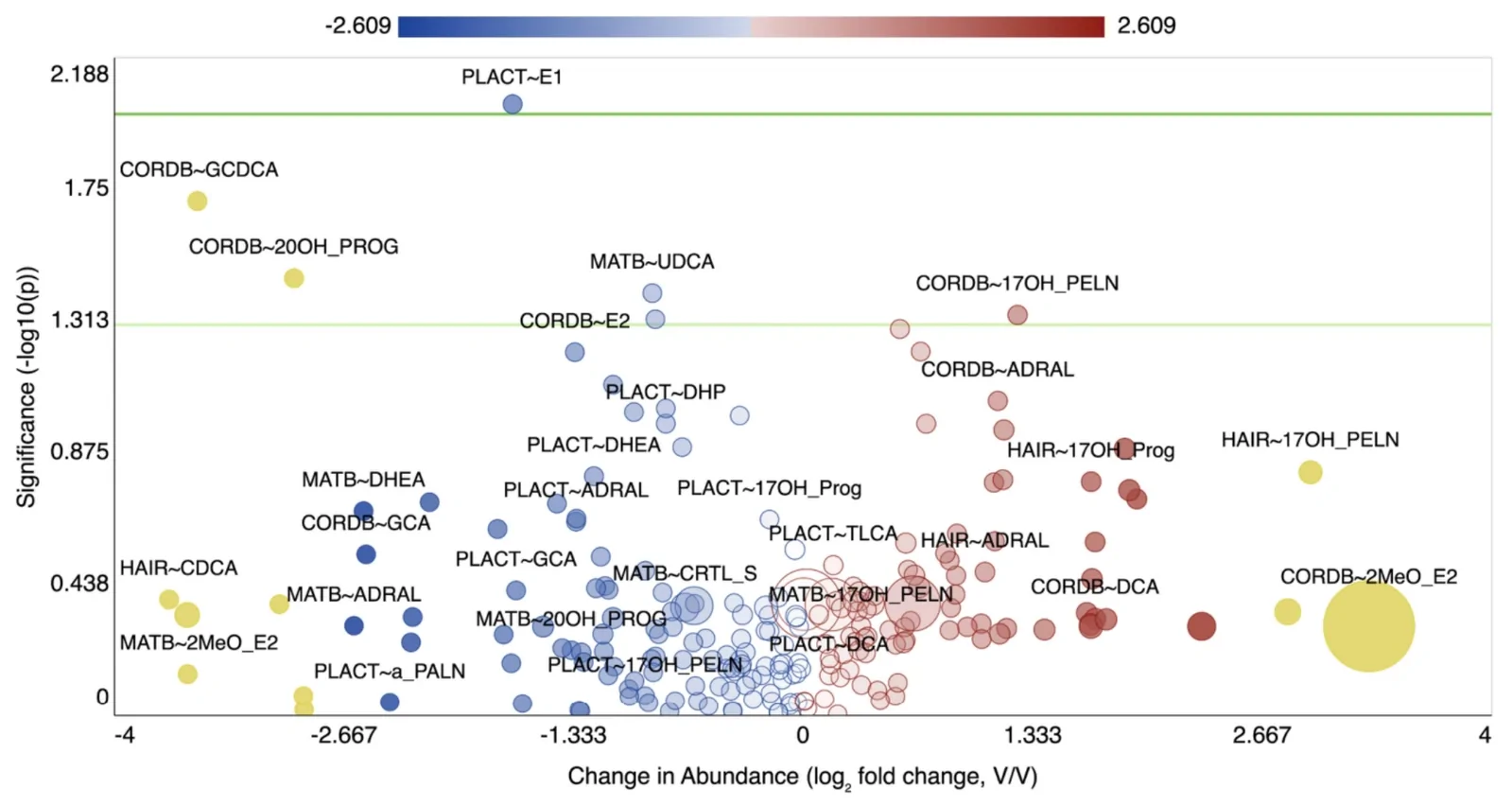
Volcano plot comparing metabolite abundance between High-PSS and Low-PSS pregnancies across maternal plasma, placental tissue, cord plasma, and maternal hair compartments. The *x*-axis represents the log_2_-fold change in metabolite abundance between groups, and the *y*-axis represents statistical significance as −log_10_ (*p*-value). Metabolites above the horizontal significance threshold (green line; *p* < 0.05, nominal, uncorrected) were considered associated with higher PSS. Placental estrone (PLACT~E1), cord plasma glycochenodeoxycholic acid (CORDB~GCDCA), and maternal plasma ursodeoxycholic acid (MATB~UDCA) were nominally (*p* < 0.05) reduced in the High-PSS group (uncorrected *p* < 0.05; none survived Benjamini–Hochberg correction). Positive fold-change values indicate metabolites elevated in High-PSS pregnancies, whereas negative values indicate metabolites reduced in High-PSS pregnancies. Bubble size reflects percent change in the metabolite abundance compared to the Low-PSS group. V/V: value-to-value.

**Figure 5:**
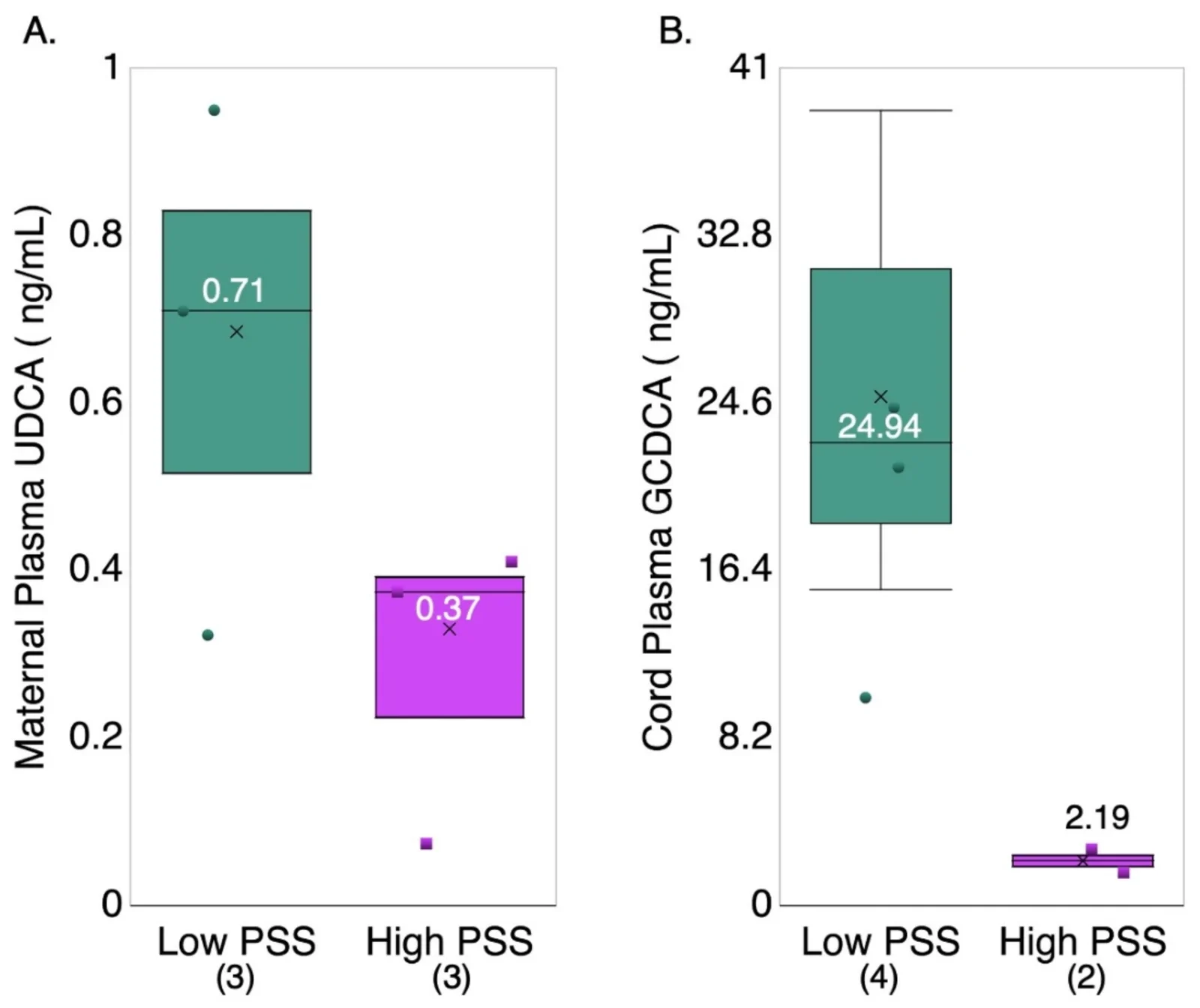
Box-and-whisker plots showing metabolites nominally associated with maternal perceived stress (PSS) change between Low- and High-PSS groups (*p* < 0.05). Two bile acid metabolites were nominally significant: (**A**) maternal plasma ursodeoxycholic acid (UDCA), and (**B**) cord plasma glycochenodeoxycholic acid (GCDCA). Individual points represent the reported analyte concentration for individual participants who provided the relevant biospecimen. Numbers in parentheses indicate the individual specimen from a given biological compartment in that group. Median values are shown. Boxes show the interquartile range with the median as the horizontal line and the mean (×); whiskers show the full range; points show individual participants’ values.

**Figure 6:**
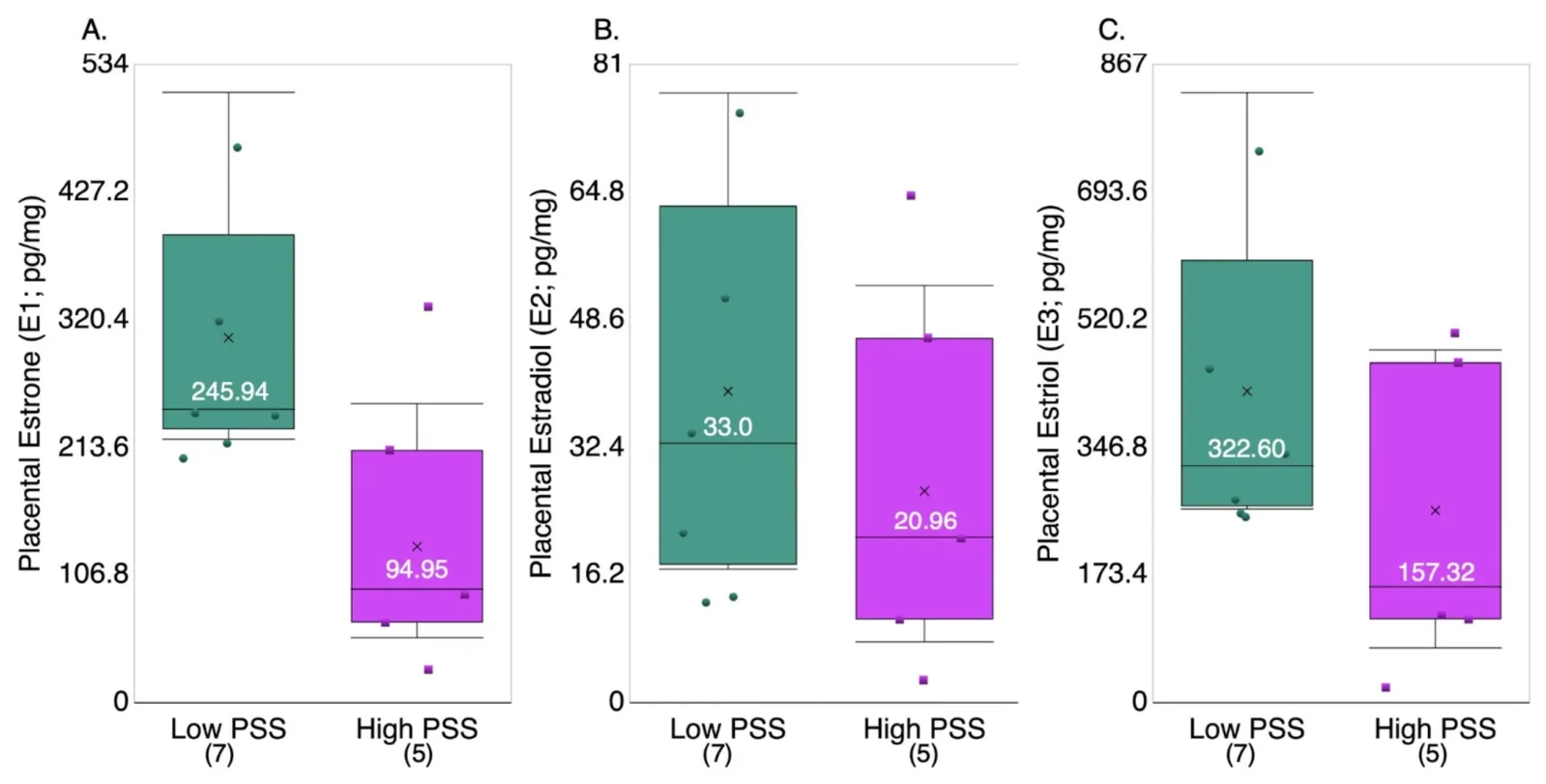
(**A**) Placental estrone (E1), (**B**) estradiol (E2), and (**C**) estriol (E3) concentrations (median; pg/mg) in Low-PSS and High-PSS pregnancies. Placental E1 abundance was nominally reduced in the High-PSS group compared with the Low-PSS group (uncorrected *p* < 0.05; did not survive multiple-testing correction), whereas placental E2 and E3 concentrations did not significantly differ between groups. Median values are shown. Boxes show the interquartile range (IQR), with the median as the horizontal line and the mean (×); whiskers show the full range; points show individual participants’ values.

**Figure 7:**
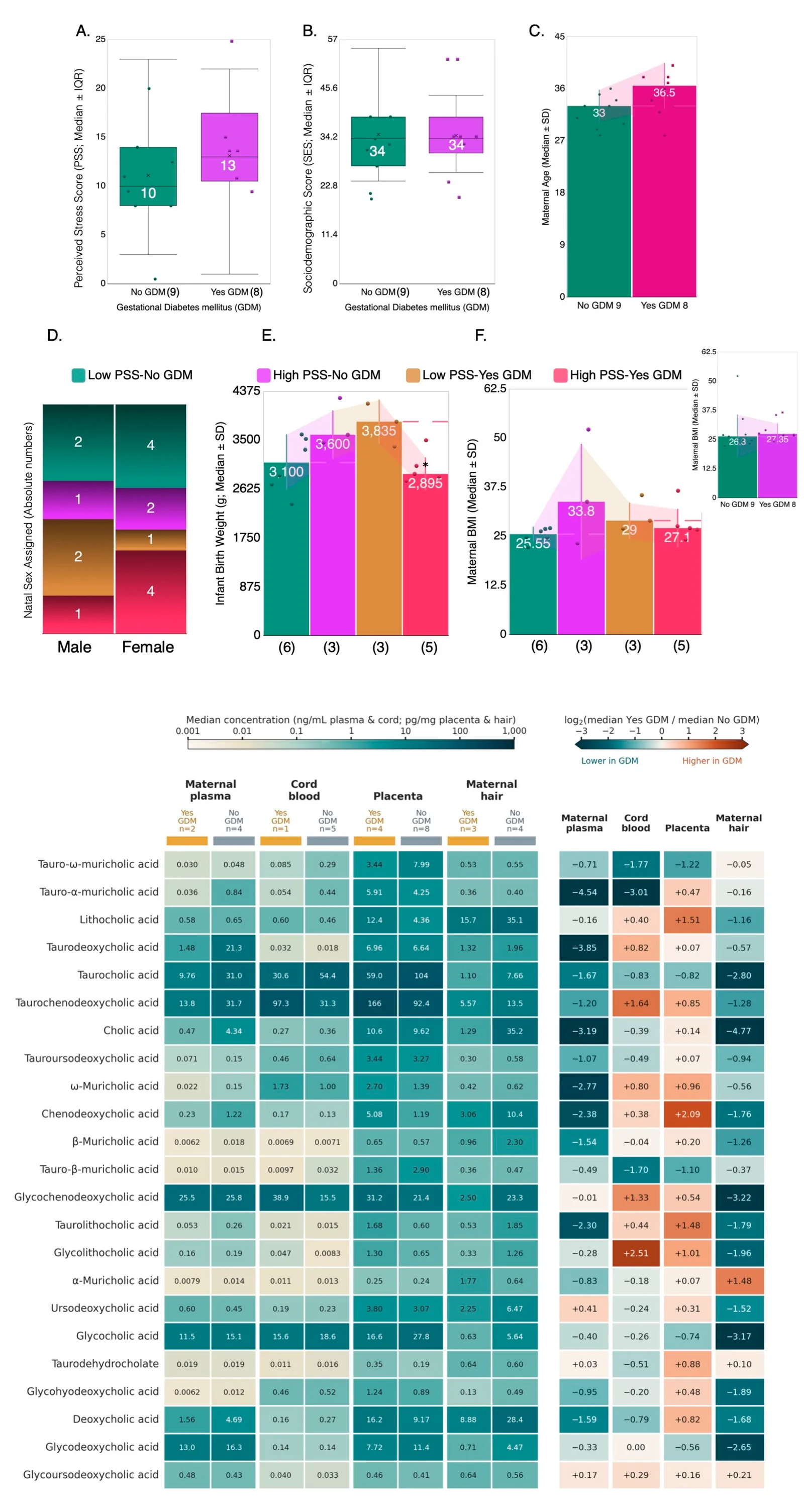

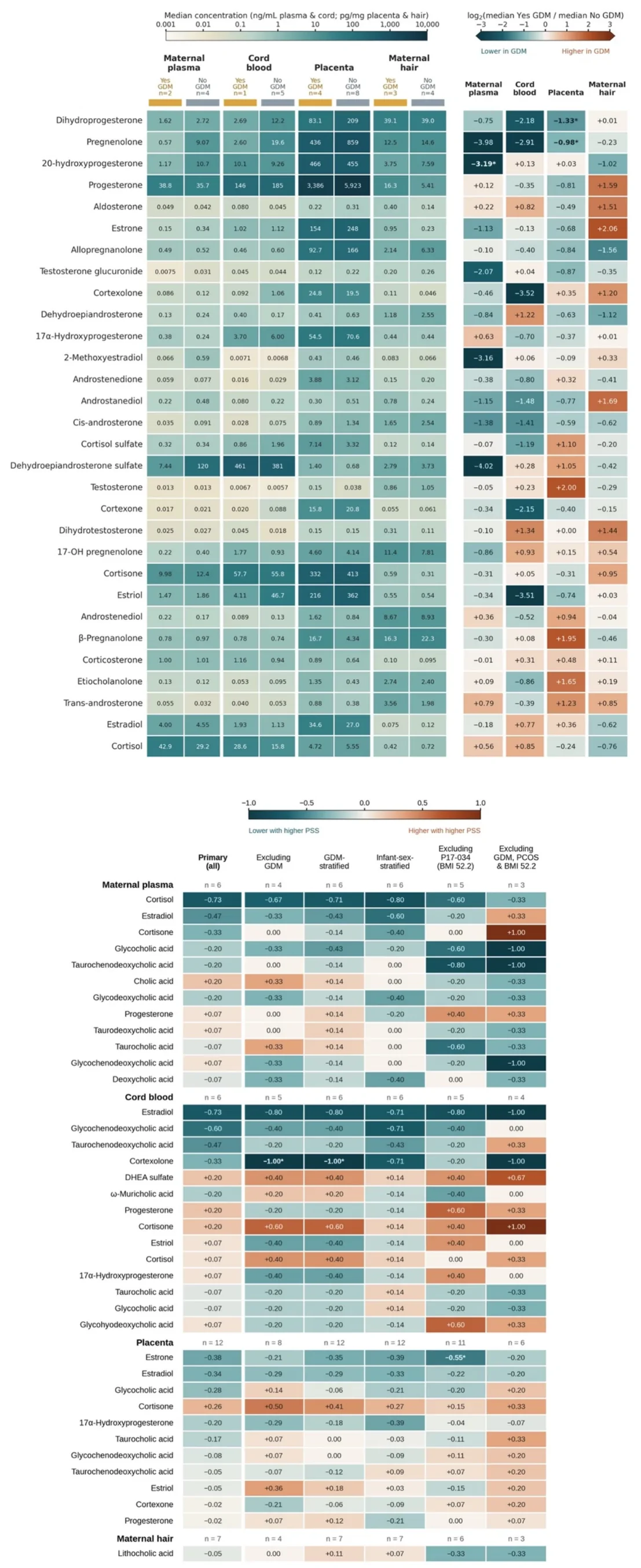
Gestational diabetes mellitus (GDM) status in relation to maternal and neonatal characteristics and metabolite profiles. (**A**,**B**) Perceived Stress Scale (PSS) and sociodemographic scores did not differ by GDM status (No GDM n = 9, median PSS = 10; Yes GDM n = 8, median PSS = 13). (**C**) Maternal age by GDM status (No GDM median = 33; Yes GDM median = 36.5 years). (**D**) Infant sex distribution across PSS × GDM subgroups. (**E**) Birth weight (g) across PSS × GDM subgroups. (**F**) Maternal BMI (kg/m^2^) across PSS × GDM subgroups (Low PSS–No GDM, n = 6, median = 25.55; High PSS–No GDM, n = 3, median = 33.8; Low PSS–Yes GDM, n = 3, median = 29; High PSS–Yes GDM, n = 5, median = 27.1); inset shows maternal BMI by GDM status alone (No GDM n = 9, median = 26.3; Yes GDM n = 8, median = 27.35). (**G**,**H**) Median bile acid (**G**) and steroid (**H**) concentrations by GDM status across four biological compartments (maternal plasma, cord plasma, placenta, maternal hair); the adjoining heatmap shows log2(median Yes-GDM/median No-GDM), with teal indicating lower and orange indicating higher concentration in GDM pregnancies. Asterisks denote nominal *p* < 0.05 (uncorrected Welch *t*-test; not surviving multiple-testing correction). (**I**) Sensitivity of the primary Kendall’s tau-b associations between continuous PSS and metabolite concentration, shown across six conditions: the full cohort (Primary, all), excluding participants with GDM, GDM-stratified, infant-sex-stratified, excluding the participant with BMI 52.2, and excluding participants with GDM, PCOS, and BMI 52.2 jointly. Color indicates the tau-b coefficient (teal, lower with higher PSS; orange, higher with higher PSS); asterisks denote nominal *p* < 0.05 (uncorrected Welch *t*-test; none survived multiple-testing correction). n reflects the number of participants contributing to each compartment under each condition.

**Table 1:** Study population characteristics. Apgar: appearance, pulse, grimace, activity, and respiration score; ART/IVF: assisted reproductive technology/in vitro fertilization; BMI: body mass index; C/S: caesarian section; BP: blood pressure; GDM/A: gestational diabetes mellitus (type A1/A2); GHTN: gestational hypertension; F: female; LTCS: lower-segment transverse cesarean section; N/A: not available/missing record; NSVD: normal spontaneous vaginal delivery; M: male; PCOS: polycystic ovary syndrome; PSS: perceived stress score; SES: sociodemographic score; VAVD: vacuum-assisted vaginal delivery.

Patient	Maternal BMI	Maternal Age	PSS	SES	Care Type	Gestational Age	Infant Weight (g)	Infant Sex	Mode of Delivery	Apgar	Notes:	Cord Plasma	Placenta	Maternal Plasma	Hair
P-002	24.8	35	11	39	Individual	39 + 4	2710	F	NSVD	99	possible Zika exposure, Rh negative, no gestational diabetes	✅	✅		
P-006	27.6	40	14	28	Centering	39 + 2	2772	F	NSVD	99	GDMA1, asthma,			✅	✅
P-016	26.3	34	10	39	Centering	39 + 6	3330	M	NSVD	9 9	chorioamnionitis, 2 elevated BPs	✅	✅	✅	✅
P-022	26.9	31	3	24	Individual	37 + 0	2360	F	NSVD	99	GHTN	✅	✅	✅	✅
P-023	23.3	33	16	33	Centering	41 + 3	3600	M	NSVD	8 9	PCOS, anemia		✅		
P-026	27	32	1	33	Individual	41 + 1	3390	M	NSVD	8 9	GDMA1, asthma,	✅	✅		
P-030	26.7	38	13	34	Individual	40 + 0	3500	F	NSVD	8 9	GDMA1, ART/IVF				✅
P-032	29	37	11	34	Centering	40 + 0	3835	F	LTCS	9 9	GDMA1, Previous C/S		✅	✅	✅
P-034	52.2	30	23	55	Individual	41 + 0	4260	F	NSVD	7 9		✅	✅	✅	✅
P-037	24.7	29	8	35	Individual	39 + 3	3525	M	VAVD	9 9					
P-038	33.8	33	12	34	Individual	41 + 6	3460	F	NSVD	8 9		✅	✅	✅	
P-041	36.6	34	21	44	Individual	39 + 6	2895	F	NSVD	4 9	GDM		✅		
P-043	27.1	36	9	30	Individual	40 + 4	3605	F	NSVD	8 9	possible Zika exposure		✅		✅
P-046	27.1	28	13	34	Individual	39 + 1	2850	F	LTCS	1 9	GDMA2				
P-048	22.3	28	8	25	Individual	39 + 6	2870	F	NSVD	4 9			✅		
P-049	24.2	38	22	44	Individual	39 + 1	3040	M	NSVD	9 9	GDMA2		✅		
P-051	35.5	36	10	26	Individual	39 + 3	4160	M	LTCS	3 8	previous C/S, GDMA2				

**Table 2:** Steroid metabolite concentrations (median) across four biological compartments.

Compartment	Analyte	Units	LOD	Total (n)	n ≥ LOD (%)	Median [Q1–Q3]
Cord plasma	17alpha-Hydroxyprogesterone (17OH-PROG)	ng/mL	0.8260	6	6 (100.0%)	5.524 [4.752–7.18]
Placenta	(17OH-PROG)	pg/mg	16.5110	12	12 (100.0%)	62.86 [49.62–77.93]
Cord plasma	Cortexolone (DXL)	ng/mL	0.8660	6	5 (83.3%)	1.039 [0.9213–1.088]
Placenta	Cortexone (DXN)	pg/mg	9.9070	12	11 (91.7%)	20.77 [15.86–22.39]
Maternal plasma	Cortisol (CRTL)	ng/mL	1.8110	6	6 (100.0%)	29.15 [24.4–54.56]
Cord plasma	Cortisol	ng/mL	1.8110	6	6 (100.0%)	20.88 [11.74–27.96]
Maternal plasma	Cortisone (CRTN)	ng/mL	0.3600	6	6 (100.0%)	10.98 [6.509–16.09]
Cord plasma	Cortisone	ng/mL	0.3600	6	6 (100.0%)	56.24 [53.53–57.44]
Placenta	Cortisone	pg/mg	7.2040	12	12 (100.0%)	371.2 [285.3–436.2]
Cord plasma	Dehydroepiandrosterone sulfate (DHEAS)	ng/mL	18.4240	6	6 (100.0%)	420.9 [360.4–492.5]
Maternal plasma	Estradiol (E2)	ng/mL	0.6800	6	6 (100.0%)	4.53 [3.05–5.244]
Cord plasma	Estradiol	ng/mL	0.6800	6	5 (83.3%)	1.157 [0.9217–1.742]
Placenta	Estradiol	pg/mg	13.6090	12	10 (83.3%)	28.26 [17.34–47.51]
Cord plasma	Estriol (E3)	ng/mL	2.8820	6	6 (100.0%)	39.04 [30.4–48.53]
Placenta	Estriol	pg/mg	57.6350	12	12 (100.0%)	301.4 [237.4–454.6]
Placenta	Estrone (E1)	pg/mg	54.0320	12	12 (100.0%)	236.9 [153.8–262.1]
Maternal plasma	Progesterone (PROG)	ng/mL	0.3930	6	6 (100.0%)	37.55 [33.72–40.38]
Cord plasma	Progesterone	ng/mL	0.3930	6	6 (100.0%)	170.8 [148.8–229.8]
Placenta	Progesterone	pg/mg	7.8560	12	12 (100.0%)	5135 [3899–6151]

**Table 3:** Bile acid metabolite concentrations across four biological compartments.

Compartment	Analyte	Units	LOD	Total (n)	n ≥ LOD (%)	Median [Q1–Q3]
Maternal plasma	Cholic acid (CA)	ng/mL	0.4083	6	5 (83.3%)	0.9222 [0.452–5.849]
Maternal plasma	Deoxycholic acid (DCA)	ng/mL	0.3923	6	5 (83.3%)	2.028 [1.074–6.274]
Maternal plasma	Glycochenodeoxycholic acid (GCDCA)	ng/mL	0.4493	6	6 (100.0%)	25.51 [16.18–35.22]
Cord plasma	Glycochenodeoxycholic acid	ng/mL	0.4493	6	6 (100.0%)	18.66 [5.726–23.1]
Placenta	Glycochenodeoxycholic acid	pg/mg	8.9863	12	12 (100.0%)	26.23 [19.82–41.99]
Maternal plasma	Glycocholic acid (GCA)	ng/mL	0.4653	6	6 (100.0%)	12.18 [10.49–17.23]
Cord plasma	Glycocholic acid	ng/mL	0.4653	6	6 (100.0%)	17.07 [14.19–25.94]
Placenta	Glycocholic acid	pg/mg	9.3062	12	10 (83.3%)	21.09 [10.99–43.52]
Maternal plasma	Glycodeoxycholic acid (GDCA)	ng/mL	0.4493	6	6 (100.0%)	14.89 [2.646–27.02]
Cord plasma	Glycohyodeoxycholic acid	ng/mL	0.4493	6	5 (83.3%)	0.493 [0.458–0.5896]
Maternal hair	Lithocholic acid (LCA)	pg/mg	7.5260	7	6 (85.7%)	16.28 [11.93–35.11]
Cord plasma	Omega-muricholic acid (ω-MCA)	ng/mL	0.4083	6	5 (83.3%)	1.051 [0.8864–1.266]
Maternal plasma	Taurochenodeoxycholic acid (TCDCA)	ng/mL	0.4993	6	6 (100.0%)	21.36 [15.74–35.77]
Cord plasma	Taurochenodeoxycholic acid	ng/mL	0.4993	6	6 (100.0%)	49.67 [31.27–68.18]
Placenta	Taurochenodeoxycholic acid	pg/mg	9.9859	12	11 (91.7%)	102.4 [64.12–162.4]
Maternal plasma	Taurocholic acid (TCA)	ng/mL	0.5153	6	6 (100.0%)	17.62 [8.841–34.62]
Cord plasma	Taurocholic acid	ng/mL	0.5153	6	6 (100.0%)	42.52 [18.81–56.5]
Placenta	Taurocholic acid	pg/mg	10.3058	12	11 (91.7%)	82.69 [43.59–111.3]
Maternal plasma	Taurodeoxycholic acid (TDCA)	ng/mL	0.4993	6	5 (83.3%)	7.041 [2.008–26.08]

## Data Availability

Data is contained within the article. LOD/LOQ, sample-matching information, and de-identified individual-level data is provided in the Supplemental Tables. Any additional information can be requested from the corresponding author and will be made available as per IRB approvals.

## Supplementary Materials

The following supporting information can be downloaded at: https://www.mdpi.com/article/10.3390/cells15211982/s1.

## Abbreviations

The following abbreviations are used in this manuscript:

20-OH-PROG	20-hydroxyprogesterone
BMI	Body mass index
BHT	Butylated hydroxytoluene
CA	Cholic acid
CDCA	Chenodeoxycholic acid
CORDB	Cord blood/plasma
CUDA	12-(3-Cyclohexylureido)dodecanoic acid
DHP	Dihydroprogesterone
E1	Estrone
E2	Estradiol
E3	Estriol
EDTA	Ethylenediaminetetraacetic acid
FXR	Farnesoid X receptor
GCDCA	Glycochenodeoxycholic acid
GDM	Gestational diabetes mellitus
HPA axis	Hypothalamic–pituitary–adrenal axis
HPLC	High-performance liquid chromatography
HSD17β1	Hydroxysteroid 17-beta dehydrogenase 1
ICP	Intrahepatic cholestasis of pregnancy
IRB	Institutional Review Board
LOD	Limit of detection
LOQ	Limit of quantification
MATB	Maternal blood/plasma
MRM	Multiple reaction monitoring
PELN	Pregnenolone
PHAU	Phenylhexadecanoic acid
PLACT	Placenta
PSS	Perceived stress scale
SES	Sociodemographic/economic score
TGR5	Takeda G-protein-coupled bile acid receptor 5 (also known as GPBAR1)
UDCA	Ursodeoxycholic acid
UPLC–MS/MS	Ultra-performance liquid chromatography with tandem mass spectrometry
