# Supplementary material for: Exploratory Multi-Compartment Profiling of Steroid and Bile Acid Metabolites in Relation to Maternal Perceived Stress at Term
Source: Cells. 2026 Oct 30;15(21):1982. doi: 10.3390/cells15211982 (PMC13762318; doi:10.3390/cells15211982)
Supplement: Supplementary file 1 [file cells-15-01982-s001.zip › cells-4481346-Supplementary Material_Junco-Gomez.pdf]

Supplementary Material for:

Exploratory Multi-Compartment Profiling of Steroid and Bile Acid Metabolites in Relation to Maternal Perceived Stress at Term

Raquel G. Junco[1], Akshay Uttarkar[1], Jeanette Lager[2] and Aditi Bhargava[1,2,*^]

[1] Aseesa Inc., South San Francisco, CA, USA 1; raquelgomezjunco@gmail.com
[2] Department of ObGyn, University of California San Francisco, San Francisco, CA, USA 2; jeanette.lager@ucsf.edu
* Correspondence: Aditi.bhargava@ucsf.edu ORCID iD: 0000-0003-1334-0517
^ Current affiliation: aditi@aseesa.com

Plasma Sample Processing

Precipitation procedure from plasma

1 Thaw plasma samples on ice
2 Vortex plasma sample and pipette 50µl in polypropylene Eppendorf tube
3 Add Surrogate Standards of the 3 lipid classes
and other reagents as in the following table:

	Volume		%	Concentration
Plasma Sample & Utak-IS 1-4	50	uL	20	
10x BA Surrogate STDs**	25	uL	10	250nM, 25nM Final
10x Ster Surrogate STDs**	25	uL	10	250nM, 25nM Final
CUDA + PHAU	25	uL	10	1000nM, 100nM Final
Anti-oxidant	25	uL	10	
ACN/MeOH	100	uL	40	
SUM	250		100	

4 Vortex for 30sec
5 Centrifuge at 6°C for 5 min at 15,000 rcf
6 Filter supernatant (~240µl) into PVDC Filter plate
7 Collect and Store in -20°C until analysis

Method Blanks	Volume		%	Concentration
Plasma Sample	0	uL	0	
10x BA Surrogate STDs**	0	uL	0	250nM, 25nM Final
10x Ster Surrogate STDs**	0	uL	0	250nM, 25nM Final
CUDA + PHAU	25	uL	10	1000nM, 100nM Final

Anti-oxidant	25	uL	10	
ACN/MeOH	200	uL	80	
SUM	250		100	

Internal Std Check	Volume		%	Concentration
Plasma Sample	0	uL	0	
10x BA Surrogate STDs**	25	uL	0	250nM, 25nM Final
10x Ster Surrogate STDs**	25	uL	0	250nM, 25nM Final
CUDA + PHAU	25	uL	10	1000nM, 100nM Final
Anti-oxidant	25	uL	10	
ACN/MeOH	150	uL	60	
SUM	250		100	

Tissue Sample Processing

	Extraction using La Frano protocol (modification)
1	Keep tissue aliquots on dry ice, record exact weights, place in 2-mL eppendorf tubes labeled on cap, keep frozen
2	Add 10 μL anti-oxidant soln, (0.2 mg/ml solution BHT/EDTA in 1:1 meoh:water)
3	Add 10 uL of EACH surrogate (SSTD, 250 nM (deuterated bile acids and steroids for recovery calculations during data processing)
4	Add 500 uL of cold methanol as extraction solvent and stainless steel grinding balls
5	Homogenize using GenoGrinder 2x30 sec, centrifuge, transfer supernatant to a 1.5-mL eppendorf tube (Cryo-label on side)
6	Add a second aliquot of 500 uL of cold methanol to centrifugation pellet
7	Homogenize using GenoGrinder 2x30 sec, centrifuge, combine this second supernatant with the first one in the 1.5-mL tube
8	Transfer vials or plate to Speed-vac and evaporate to dryness
9	Assure samples are dry before taking out of Speed-vac.
10	Reconstitute samples for LC-MS in 100 uL of PHAU/CUDA 100 nM in methanol/ACN 50:50, vortex for 10 sec, then sonicate for 5 min.
11	Set rack of samples on wet ice for 15min
12	Centrifuge for 3 min at highest speed, then transfer supernatant to PVDC filter plate and centrifuge for 6 min
13	Store filtrate at -20°C until LCMS analysis
	Analyze by LC/MS on Waters UPLC/AB Sciex Qtrap with targeted MRM method (developed by Newman lab, optimized for Fiehn lab setup)
	Re-cap and store at -20°C for potential re-analysis

Supplementary Figure S1.

Fig. S1

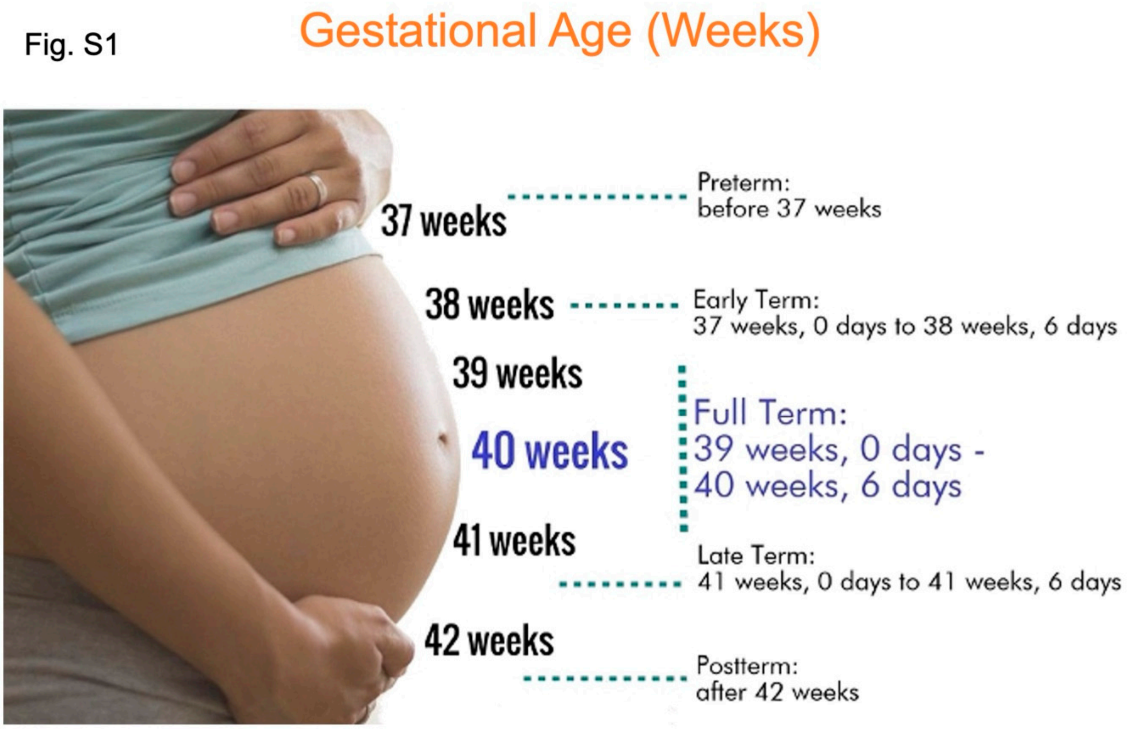

https://www.acog.org/About-ACOG/ACOG-Districts/District-II/Preventing-Preterm-Birth-for-Patients

Figure S1 Legend. Gestation age (weeks) in human pregnancy. Birth before 37 weeks of gestational age is considered preterm, whereas as full-term pregnancy ranges between 39 weeks, 0 days to 40 weeks, 6 days.

Fig. S2

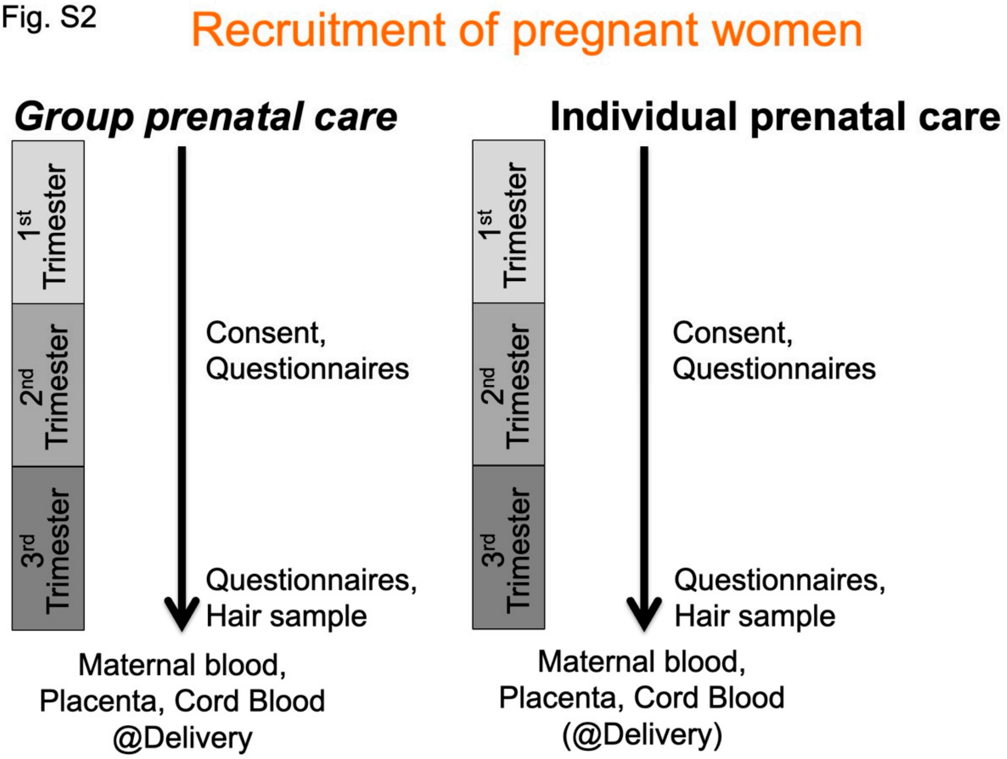

Figure S2. Legend. Recruitment timeline for pregnant women at UCSF.

Fig. S3

The Placenta

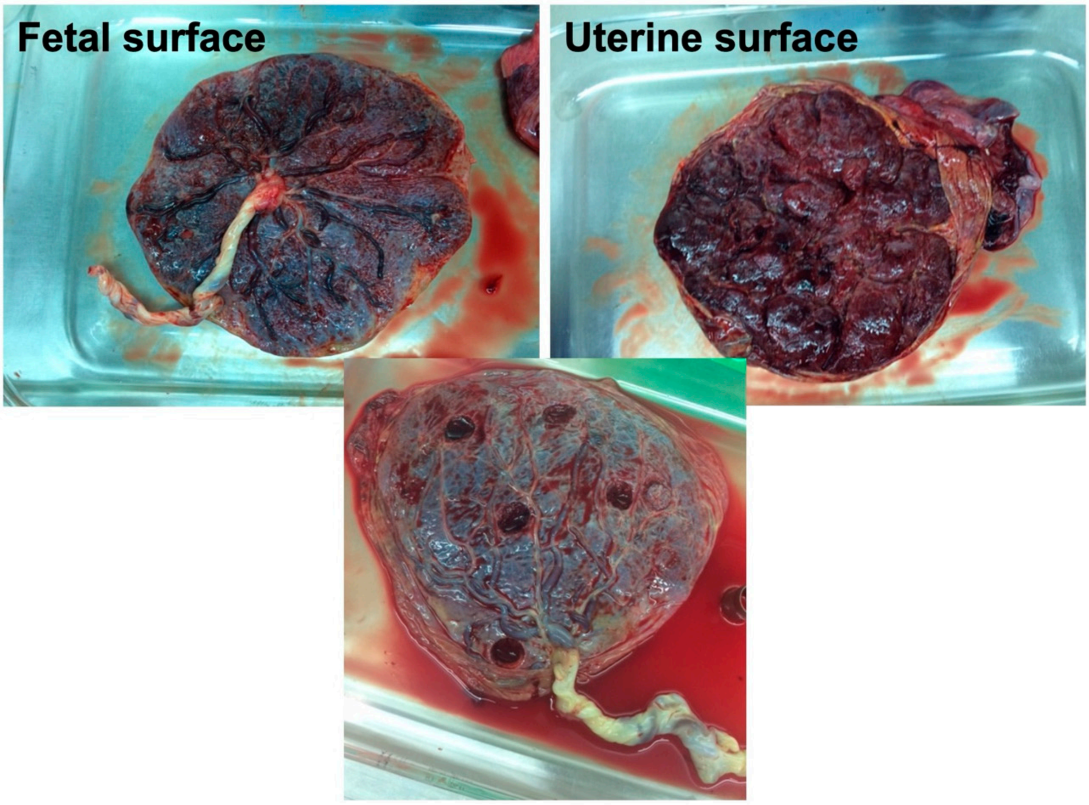

Figure S3 Legend. Placenta collected after delivery. Placental tissue was sampled by punching five biopsies through the full thickness of the placenta, from the uterine to the fetal surface. This sampling pattern was applied consistently across all placentae to ensure representative coverage of different regions.

Figure S4. Mother-Infant Demographics

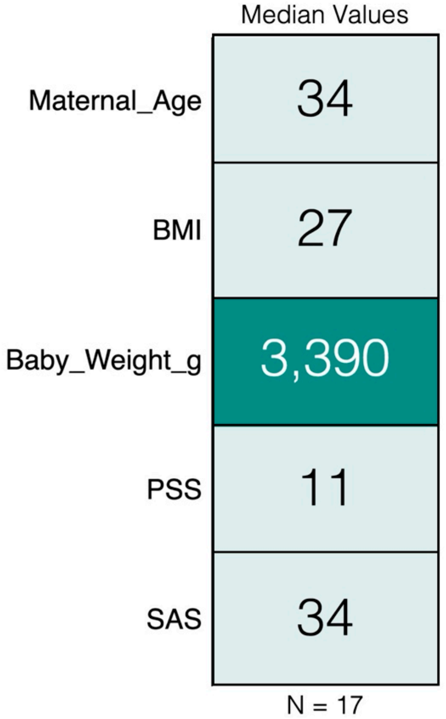

Figure S4. Legend. Anthropometeric statistics for mother-infant features. PSS scores ranged from 1 to 23 (median 11.0; IQR: 4.99 – 17.00), socioeconomic scores ranged from 25 to 55 (median 34; IQR: 26.17 – 41.83), maternal BMI ranged from 22.3 to 52.2 (median 27.0; IQR: 19.81 – 34.19), maternal age ranged from 29 to 40 years (median 34 years; IQR: 30.36 – 37.63), and infant birth weight ranged from 2,360 g to 4,260 g (median 3,390 g; IQR: 2,866.49 – 3,913.50).

Figure S5. Maternal BMI and Age by Care and PSS groups

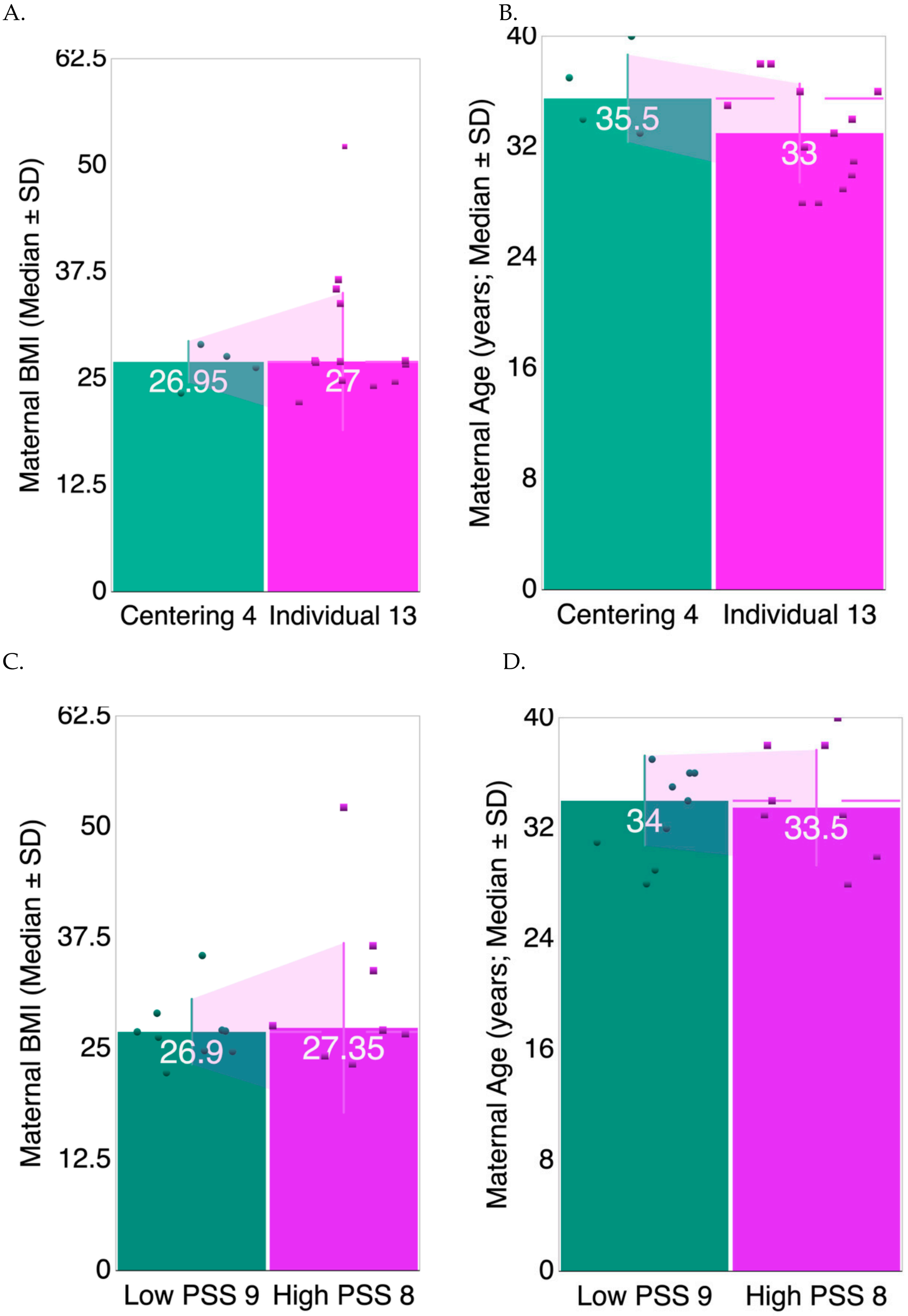

Figure S5 Legend. Maternal BMI and age (Median) did not differ between care (centering versus individual) and low versus high PSS split.
