# Supplementary material for: Exploratory Multi-Compartment Profiling of Steroid and Bile Acid Metabolites in Relation to Maternal Perceived Stress at Term
Source: Cells. 2026 Oct 30;15(21):1982. doi: 10.3390/cells15211982 (PMC13762318; doi:10.3390/cells15211982)
Supplement: Supplementary file 1 [file cells-15-01982-s001.zip › cells-4481346-TableS2_LOD_values.pdf]

Supplementary Table S2.

Analyte class	Analyte	Matrix	Unit	Total n	n ≥LOD	Detection frequency
Bile acid	Alpha-muricholic acid	Maternal plasma	ng/mL	6	0	0.0%
Bile acid	Alpha-muricholic acid	Cord blood	ng/mL	6	0	0.0%
Bile acid	Alpha-muricholic acid	Placenta	pg/mg	12	0	0.0%
Bile acid	Alpha-muricholic acid	Maternal hair	pg/mg	7	0	0.0%
Bile acid	Beta-muricholic acid	Maternal plasma	ng/mL	6	0	0.0%
Bile acid	Beta-muricholic acid	Cord blood	ng/mL	6	0	0.0%
Bile acid	Beta-muricholic acid	Placenta	pg/mg	12	0	0.0%
Bile acid	Beta-muricholic acid	Maternal hair	pg/mg	7	0	0.0%
Bile acid	Chenodeoxycholic acid	Maternal plasma	ng/mL	6	4	66.7%
Bile acid	Chenodeoxycholic acid	Cord blood	ng/mL	6	1	16.7%
Bile acid	Chenodeoxycholic acid	Placenta	pg/mg	12	4	33.3%
Bile acid	Chenodeoxycholic acid	Maternal hair	pg/mg	7	4	57.1%
Bile acid	Cholic acid	Maternal plasma	ng/mL	6	5	83.3%
Bile acid	Cholic acid	Cord blood	ng/mL	6	1	16.7%
Bile acid	Cholic acid	Placenta	pg/mg	12	6	50.0%
Bile acid	Cholic acid	Maternal hair	pg/mg	7	3	42.9%
Bile acid	Deoxycholic acid	Maternal plasma	ng/mL	6	5	83.3%
Bile acid	Deoxycholic acid	Cord blood	ng/mL	6	1	16.7%
Bile acid	Deoxycholic acid	Placenta	pg/mg	12	6	50.0%
Bile acid	Deoxycholic acid	Maternal hair	pg/mg	7	5	71.4%
Bile acid	Glycochenodeoxycholic acid	Maternal plasma	ng/mL	6	6	100.0%
Bile acid	Glycochenodeoxycholic acid	Cord blood	ng/mL	6	6	100.0%
Bile acid	Glycochenodeoxycholic acid	Placenta	pg/mg	12	12	100.0%
Bile acid	Glycochenodeoxycholic acid	Maternal hair	pg/mg	7	2	28.6%

Bile acid	Glycocholic acid	Maternal plasma	ng/mL	6	6	100.0%
Bile acid	Glycocholic acid	Cord blood	ng/mL	6	6	100.0%
Bile acid	Glycocholic acid	Placenta	pg/mg	12	10	83.3%
Bile acid	Glycocholic acid	Maternal hair	pg/mg	7	2	28.6%
Bile acid	Glycodeoxycholic acid	Maternal plasma	ng/mL	6	6	100.0%
Bile acid	Glycodeoxycholic acid	Cord blood	ng/mL	6	0	0.0%
Bile acid	Glycodeoxycholic acid	Placenta	pg/mg	12	7	58.3%
Bile acid	Glycodeoxycholic acid	Maternal hair	pg/mg	7	2	28.6%
Bile acid	Glycohyodeoxycholic acid	Maternal plasma	ng/mL	6	0	0.0%
Bile acid	Glycohyodeoxycholic acid	Cord blood	ng/mL	6	5	83.3%
Bile acid	Glycohyodeoxycholic acid	Placenta	pg/mg	12	0	0.0%
Bile acid	Glycohyodeoxycholic acid	Maternal hair	pg/mg	7	0	0.0%
Bile acid	Glycolithocholic acid	Maternal plasma	ng/mL	6	1	16.7%
Bile acid	Glycolithocholic acid	Cord blood	ng/mL	6	0	0.0%
Bile acid	Glycolithocholic acid	Placenta	pg/mg	12	0	0.0%
Bile acid	Glycolithocholic acid	Maternal hair	pg/mg	7	0	0.0%
Bile acid	Glycoursodeoxycholic acid	Maternal plasma	ng/mL	6	3	50.0%
Bile acid	Glycoursodeoxycholic acid	Cord blood	ng/mL	6	0	0.0%
Bile acid	Glycoursodeoxycholic acid	Placenta	pg/mg	12	0	0.0%
Bile acid	Glycoursodeoxycholic acid	Maternal hair	pg/mg	7	0	0.0%
Bile acid	Lithocholic acid	Maternal plasma	ng/mL	6	4	66.7%
Bile acid	Lithocholic acid	Cord blood	ng/mL	6	4	66.7%
Bile acid	Lithocholic acid	Placenta	pg/mg	12	7	58.3%
Bile acid	Lithocholic acid	Maternal hair	pg/mg	7	6	85.7%
Bile acid	Omega-muricholic acid	Maternal plasma	ng/mL	6	0	0.0%
Bile acid	Omega-muricholic acid	Cord blood	ng/mL	6	5	83.3%
Bile acid	Omega-muricholic acid	Placenta	pg/mg	12	0	0.0%

Bile acid	Omega-muricholic acid	Maternal hair	pg/mg	7	0	0.0%
Bile acid	Tauro-alpha-muricholic acid	Maternal plasma	ng/mL	6	2	33.3%
Bile acid	Tauro-alpha-muricholic acid	Cord blood	ng/mL	6	1	16.7%
Bile acid	Tauro-alpha-muricholic acid	Placenta	pg/mg	12	4	33.3%
Bile acid	Tauro-alpha-muricholic acid	Maternal hair	pg/mg	7	0	0.0%
Bile acid	Tauro-beta-muricholic acid	Maternal plasma	ng/mL	6	0	0.0%
Bile acid	Tauro-beta-muricholic acid	Cord blood	ng/mL	6	0	0.0%
Bile acid	Tauro-beta-muricholic acid	Placenta	pg/mg	12	1	8.3%
Bile acid	Tauro-beta-muricholic acid	Maternal hair	pg/mg	7	0	0.0%
Bile acid	Tauro-omega-muricholic acid	Maternal plasma	ng/mL	6	0	0.0%
Bile acid	Tauro-omega-muricholic acid	Cord blood	ng/mL	6	1	16.7%
Bile acid	Tauro-omega-muricholic acid	Placenta	pg/mg	12	3	25.0%
Bile acid	Tauro-omega-muricholic acid	Maternal hair	pg/mg	7	0	0.0%
Bile acid	Taurochenodeoxycholic acid	Maternal plasma	ng/mL	6	6	100.0%
Bile acid	Taurochenodeoxycholic acid	Cord blood	ng/mL	6	6	100.0%
Bile acid	Taurochenodeoxycholic acid	Placenta	pg/mg	12	11	91.7%
Bile acid	Taurochenodeoxycholic acid	Maternal hair	pg/mg	7	3	42.9%
Bile acid	Taurocholic acid	Maternal plasma	ng/mL	6	6	100.0%
Bile acid	Taurocholic acid	Cord blood	ng/mL	6	6	100.0%
Bile acid	Taurocholic acid	Placenta	pg/mg	12	11	91.7%
Bile acid	Taurocholic acid	Maternal hair	pg/mg	7	2	28.6%
Bile acid	Taurodehydrocholate	Maternal plasma	ng/mL	6	0	0.0%
Bile acid	Taurodehydrocholate	Cord blood	ng/mL	6	0	0.0%
Bile acid	Taurodehydrocholate	Placenta	pg/mg	12	1	8.3%
Bile acid	Taurodehydrocholate	Maternal hair	pg/mg	7	0	0.0%

Bile acid	Taurodeoxycholic acid	Maternal plasma	ng/mL	6	5	83.3%
Bile acid	Taurodeoxycholic acid	Cord blood	ng/mL	6	0	0.0%
Bile acid	Taurodeoxycholic acid	Placenta	pg/mg	12	3	25.0%
Bile acid	Taurodeoxycholic acid	Maternal hair	pg/mg	7	0	0.0%
Bile acid	Taurolithocholic acid	Maternal plasma	ng/mL	6	1	16.7%
Bile acid	Taurolithocholic acid	Cord blood	ng/mL	6	0	0.0%
Bile acid	Taurolithocholic acid	Placenta	pg/mg	12	0	0.0%
Bile acid	Taurolithocholic acid	Maternal hair	pg/mg	7	0	0.0%
Bile acid	Tauroursodeoxycholic acid	Maternal plasma	ng/mL	6	0	0.0%
Bile acid	Tauroursodeoxycholic acid	Cord blood	ng/mL	6	4	66.7%
Bile acid	Tauroursodeoxycholic acid	Placenta	pg/mg	12	4	33.3%
Bile acid	Tauroursodeoxycholic acid	Maternal hair	pg/mg	7	0	0.0%
Bile acid	Ursodeoxycholic acid	Maternal plasma	ng/mL	6	3	50.0%
Bile acid	Ursodeoxycholic acid	Cord blood	ng/mL	6	0	0.0%
Bile acid	Ursodeoxycholic acid	Placenta	pg/mg	12	1	8.3%
Bile acid	Ursodeoxycholic acid	Maternal hair	pg/mg	7	2	28.6%
Steroid	17-OH pregnenolone	Maternal plasma	ng/mL	6	0	0.0%
Steroid	17-OH pregnenolone	Cord blood	ng/mL	6	0	0.0%
Steroid	17-OH pregnenolone	Placenta	pg/mg	12	0	0.0%
Steroid	17-OH pregnenolone	Maternal hair	pg/mg	7	0	0.0%
Steroid	17alpha-Hydroxyprogesterone	Maternal plasma	ng/mL	6	1	16.7%
Steroid	17alpha-Hydroxyprogesterone	Cord blood	ng/mL	6	6	100.0%
Steroid	17alpha-Hydroxyprogesterone	Placenta	pg/mg	12	12	100.0%
Steroid	17alpha-Hydroxyprogesterone	Maternal hair	pg/mg	7	0	0.0%
Steroid	2-methoxyestradiol	Maternal plasma	ng/mL	6	2	33.3%
Steroid	2-methoxyestradiol	Cord blood	ng/mL	6	1	16.7%

Steroid	2-methoxyestradiol	Placenta	pg/mg	12	0	0.0%
Steroid	2-methoxyestradiol	Maternal hair	pg/mg	7	0	0.0%
Steroid	20-hydroxyprogesterone	Maternal plasma	ng/mL	6	0	0.0%
Steroid	20-hydroxyprogesterone	Cord blood	ng/mL	6	0	0.0%
Steroid	20-hydroxyprogesterone	Placenta	pg/mg	12	0	0.0%
Steroid	20-hydroxyprogesterone	Maternal hair	pg/mg	7	0	0.0%
Steroid	Aldosterone	Maternal plasma	ng/mL	6	0	0.0%
Steroid	Aldosterone	Cord blood	ng/mL	6	0	0.0%
Steroid	Aldosterone	Placenta	pg/mg	12	0	0.0%
Steroid	Aldosterone	Maternal hair	pg/mg	7	0	0.0%
Steroid	Allopregnanolone	Maternal plasma	ng/mL	6	1	16.7%
Steroid	Allopregnanolone	Cord blood	ng/mL	6	0	0.0%
Steroid	Allopregnanolone	Placenta	pg/mg	12	7	58.3%
Steroid	Allopregnanolone	Maternal hair	pg/mg	7	0	0.0%
Steroid	Androstanediol	Maternal plasma	ng/mL	6	0	0.0%
Steroid	Androstanediol	Cord blood	ng/mL	6	0	0.0%
Steroid	Androstanediol	Placenta	pg/mg	12	0	0.0%
Steroid	Androstanediol	Maternal hair	pg/mg	7	0	0.0%
Steroid	Androstenediol	Maternal plasma	ng/mL	6	0	0.0%
Steroid	Androstenediol	Cord blood	ng/mL	6	0	0.0%
Steroid	Androstenediol	Placenta	pg/mg	12	0	0.0%
Steroid	Androstenediol	Maternal hair	pg/mg	7	0	0.0%
Steroid	Androstenedione	Maternal plasma	ng/mL	6	0	0.0%
Steroid	Androstenedione	Cord blood	ng/mL	6	0	0.0%
Steroid	Androstenedione	Placenta	pg/mg	12	0	0.0%
Steroid	Androstenedione	Maternal hair	pg/mg	7	0	0.0%
Steroid	Beta-pregnanolone	Maternal plasma	ng/mL	6	0	0.0%
Steroid	Beta-pregnanolone	Cord blood	ng/mL	6	0	0.0%

Steroid	Beta-pregnanolone	Placenta	pg/mg	12	0	0.0%
Steroid	Beta-pregnanolone	Maternal hair	pg/mg	7	0	0.0%
Steroid	Cis-androsterone	Maternal plasma	ng/mL	6	0	0.0%
Steroid	Cis-androsterone	Cord blood	ng/mL	6	0	0.0%
Steroid	Cis-androsterone	Placenta	pg/mg	12	0	0.0%
Steroid	Cis-androsterone	Maternal hair	pg/mg	7	0	0.0%
Steroid	Cortexolone	Maternal plasma	ng/mL	6	1	16.7%
Steroid	Cortexolone	Cord blood	ng/mL	6	5	83.3%
Steroid	Cortexolone	Placenta	pg/mg	12	9	75.0%
Steroid	Cortexolone	Maternal hair	pg/mg	7	0	0.0%
Steroid	Cortexone	Maternal plasma	ng/mL	6	0	0.0%
Steroid	Cortexone	Cord blood	ng/mL	6	1	16.7%
Steroid	Cortexone	Placenta	pg/mg	12	11	91.7%
Steroid	Cortexone	Maternal hair	pg/mg	7	0	0.0%
Steroid	Corticosterone	Maternal plasma	ng/mL	6	4	66.7%
Steroid	Corticosterone	Cord blood	ng/mL	6	4	66.7%
Steroid	Corticosterone	Placenta	pg/mg	12	0	0.0%
Steroid	Corticosterone	Maternal hair	pg/mg	7	0	0.0%
Steroid	Cortisol	Maternal plasma	ng/mL	6	6	100.0%
Steroid	Cortisol	Cord blood	ng/mL	6	6	100.0%
Steroid	Cortisol	Placenta	pg/mg	12	0	0.0%
Steroid	Cortisol	Maternal hair	pg/mg	7	0	0.0%
Steroid	Cortisol sulfate	Maternal plasma	ng/mL	6	1	16.7%
Steroid	Cortisol sulfate	Cord blood	ng/mL	6	0	0.0%
Steroid	Cortisol sulfate	Placenta	pg/mg	12	0	0.0%
Steroid	Cortisol sulfate	Maternal hair	pg/mg	7	0	0.0%
Steroid	Cortisone	Maternal plasma	ng/mL	6	6	100.0%
Steroid	Cortisone	Cord blood	ng/mL	6	6	100.0%

Steroid	Cortisone	Placenta	pg/mg	12	12	100.0%
Steroid	Cortisone	Maternal hair	pg/mg	7	0	0.0%
Steroid	Dehydroepiandrosterone	Maternal plasma	ng/mL	6	0	0.0%
Steroid	Dehydroepiandrosterone	Cord blood	ng/mL	6	0	0.0%
Steroid	Dehydroepiandrosterone	Placenta	pg/mg	12	0	0.0%
Steroid	Dehydroepiandrosterone	Maternal hair	pg/mg	7	0	0.0%
Steroid	Dehydroepiandrosterone sulfate	Maternal plasma	ng/mL	6	3	50.0%
Steroid	Dehydroepiandrosterone sulfate	Cord blood	ng/mL	6	6	100.0%
Steroid	Dehydroepiandrosterone sulfate	Placenta	pg/mg	12	0	0.0%
Steroid	Dehydroepiandrosterone sulfate	Maternal hair	pg/mg	7	0	0.0%
Steroid	Dihydroprogesterone	Maternal plasma	ng/mL	6	0	0.0%
Steroid	Dihydroprogesterone	Cord blood	ng/mL	6	0	0.0%
Steroid	Dihydroprogesterone	Placenta	pg/mg	12	0	0.0%
Steroid	Dihydroprogesterone	Maternal hair	pg/mg	7	0	0.0%
Steroid	Dihydrotestosterone	Maternal plasma	ng/mL	6	0	0.0%
Steroid	Dihydrotestosterone	Cord blood	ng/mL	6	0	0.0%
Steroid	Dihydrotestosterone	Placenta	pg/mg	12	0	0.0%
Steroid	Dihydrotestosterone	Maternal hair	pg/mg	7	0	0.0%
Steroid	Estradiol	Maternal plasma	ng/mL	6	6	100.0%
Steroid	Estradiol	Cord blood	ng/mL	6	5	83.3%
Steroid	Estradiol	Placenta	pg/mg	12	10	83.3%
Steroid	Estradiol	Maternal hair	pg/mg	7	0	0.0%
Steroid	Estriol	Maternal plasma	ng/mL	6	1	16.7%
Steroid	Estriol	Cord blood	ng/mL	6	6	100.0%
Steroid	Estriol	Placenta	pg/mg	12	12	100.0%
Steroid	Estriol	Maternal hair	pg/mg	7	0	0.0%
Steroid	Estrone	Maternal plasma	ng/mL	6	1	16.7%

Steroid	Estrone	Cord blood	ng/mL	6	1	16.7%
Steroid	Estrone	Placenta	pg/mg	12	12	100.0%
Steroid	Estrone	Maternal hair	pg/mg	7	0	0.0%
Steroid	Etiocholanolone	Maternal plasma	ng/mL	6	0	0.0%
Steroid	Etiocholanolone	Cord blood	ng/mL	6	0	0.0%
Steroid	Etiocholanolone	Placenta	pg/mg	12	0	0.0%
Steroid	Etiocholanolone	Maternal hair	pg/mg	7	0	0.0%
Steroid	Pregnenolone	Maternal plasma	ng/mL	6	0	0.0%
Steroid	Pregnenolone	Cord blood	ng/mL	6	0	0.0%
Steroid	Pregnenolone	Placenta	pg/mg	12	0	0.0%
Steroid	Pregnenolone	Maternal hair	pg/mg	7	0	0.0%
Steroid	Progesterone	Maternal plasma	ng/mL	6	6	100.0%
Steroid	Progesterone	Cord blood	ng/mL	6	6	100.0%
Steroid	Progesterone	Placenta	pg/mg	12	12	100.0%
Steroid	Progesterone	Maternal hair	pg/mg	7	4	57.1%
Steroid	Testosterone	Maternal plasma	ng/mL	6	0	0.0%
Steroid	Testosterone	Cord blood	ng/mL	6	0	0.0%
Steroid	Testosterone	Placenta	pg/mg	12	0	0.0%
Steroid	Testosterone	Maternal hair	pg/mg	7	1	14.3%
Steroid	Testosterone glucuronide	Maternal plasma	ng/mL	6	0	0.0%
Steroid	Testosterone glucuronide	Cord blood	ng/mL	6	0	0.0%
Steroid	Testosterone glucuronide	Placenta	pg/mg	12	0	0.0%
Steroid	Testosterone glucuronide	Maternal hair	pg/mg	7	0	0.0%
Steroid	Trans-androsterone	Maternal plasma	ng/mL	6	0	0.0%
Steroid	Trans-androsterone	Cord blood	ng/mL	6	0	0.0%
Steroid	Trans-androsterone	Placenta	pg/mg	12	0	0.0%
Steroid	Trans-androsterone	Maternal hair	pg/mg	7	0	0.0%
